# Hydrophobic Microfibrillated Cellulose Aerogels via Dual-Silane Modification for Sustainable Oil Spill Recovery

**DOI:** 10.1021/acs.langmuir.6c03349

**Published:** 2026-10-05

**Authors:** Ninoska Isabel Bojorge Ramirez, Isabela Rosa Marchette, Bárbara Cristina Rodrigues, Eliana Mosse Alhadeff, Marta Cecilia Tapia Reyes, Ruan Stevan de Almeida Ribeiro, Troner Assenheimer de Souza, Juan Manuel Pardal, Felipe Silva Semaan

**Affiliations:** † Department of Chemical and Petroleum Engineering, Fluminense Federal University, R. Passo da Pátria, 156 − Sala 235 − Bloco E, São Domingos, Niterói 24210-240, RJ, Brazil; ‡ Department of Biochemical Engineering, 28125 Federal University of Rio de Janeiro, Sala 3, Av. Horácio Macedo, 2030, Cidade Universitária − Ilha do Fundão, Rio de Janeiro 21941-909, Brazil; § Department of Naval Engineering, 28125 Federal University of Rio de Janeiro, Av. Athos Silveira, 149 − CT − Bl, C - Sala 207, Cidade Universitária − Ilha do Fundão, Rio de Janeiro 21945-970, Brazil; ∥ Department of Mechanical Engineering, Fluminense Federal University, Rua Passo da Pátria, 156 Bl. D, Campus da Praia Vermelha, Niterói 24210-240, RJ, Brazil; ⊥ Department of Analytical Chemistry, Fluminense Federal University, Outeiro São João Batista, sn, Campus do Valonguinho, Centro, Niterói 24020-140, RJ, Brazil

## Abstract

Oil spills in marine waters threaten sensitive ecosystems. This highlights the need for effective cleanup methods. While cellulose-based aerogels absorb oil effectively, improving their water resistance without compromising their eco-friendliness remains essential. This study aims to produce hydrophobic aerogels by combining methyltrimethoxysilane (MTMS) and octadecyltrimethoxysilane (OTMS) to create a hierarchical surface with low surface energy and high hydrophobicity. The goal is to modify them and compare their performance with bleached and brown microfibrillated cellulose (MFC). The resulting aerogels have water contact angles of 133.68° ± 1.16° and show excellent selective oil absorption. The bleached MFC aerogel has absorption capacities of 20.2 g g^–1^ for cooking oil, 15.6 g g^–1^ for diesel, and 13.3 g g^–1^ for petroleum, with low water absorption of 5.2 g g^–1^. In comparison, the brown MFC aerogel performs similarly, absorbing 17.1 g g^–1^ for cooking oil, 14.9 g g^–1^ for diesel, and 12.4 g g^–1^ for petroleum, with even lower water absorption (0.5 g g^–1^). These renewable MFC aerogels selectively take up oil from oil–water systems and provide a laboratory-scale basis for further evaluation in remediation settings such as Guanabara Bay.

## Graphical abstract

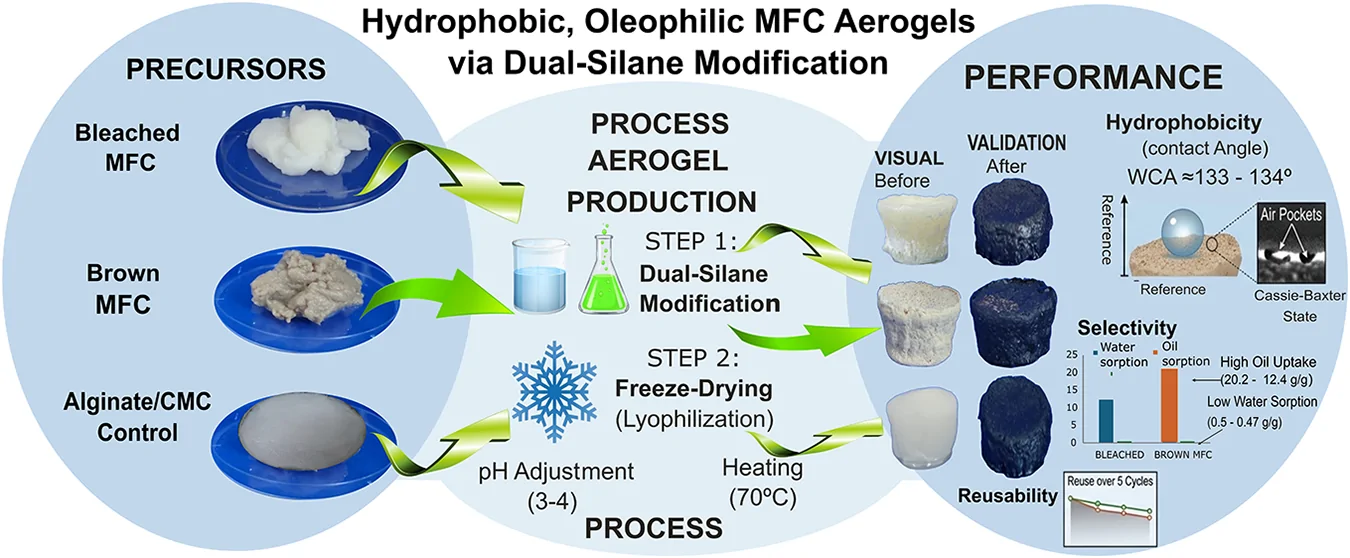

## Introduction

Oil spills in marine waters are among the most significant environmental threats to aquatic ecosystems globally. The persistence of hydrocarbons in the environment, combined with their bioaccumulation, causes long-term ecological damage that extends far beyond the immediate spill event. This urgency has prompted intensive research into effective remediation technologies.^1–3^

Guanabara Bay in Rio de Janeiro, Brazil, exemplifies this critical environmental challenge. Recent environmental sensitivity mapping has classified 89% of the bay’s 981.5 km coastline as ESI 8–10,^4^ indicating extreme vulnerability to oil impacts.^5^ The bay sustains 11 million people and 10,000 artisanal fishers producing 500 tons of fish monthly. The 2000 Petrobras oil spill (>1 million liters) resulted in months-long bans on fish consumption and documented declines in commercial species. Current chronic pollution includes 98 tons of daily waste discharge and >1.3 billion liters of wastewater, creating an urgent need for innovative remediation solutions.^6,5,6^

Untreated oily wastewater threatens the environment and human health. It wastes energy and causes economic losses.^7,8^ Releasing untreated or partially treated oil refinery wastewater pollutes water bodies. This leads to serious ecological problems, high cleanup costs, and the loss of farmland and fish resources.^9^ The composition of refinery wastewater depends on the quality of the crude oil being processed and changes with operating conditions. Refinery processes remove non-hydrocarbon elements, break down crude oil into its parts, and turn them into usable products. Oil refining plants generate large amounts of wastewater, especially those involved in oil drilling. This wastewater often contains hard-to-remove chemicals and high levels of organic pollutants. This makes treatment, especially biological treatment, quite challenging.^10^

Several remediation approaches exist, including chemical (combustion, flocculation), biological (microbial decomposition), and physical (sorption) techniques. However, chemical treatments risk secondary contamination, biological processes are slow, and traditional aerogels (carbon, silica) suffer from brittleness and poor mechanical stability. Natural polymer-based aerogels offer superior sustainability and cost-effectiveness. Cellulose represents abundant, renewable, and environmentally friendly material for aerogel production. Cellulose-based aerogels exhibit high porosity, low density, and large specific surface areas, enabling high sorption rates.^11^ Recent studies on cellulose-based biomaterials have demonstrated the potential of nanocellulose aerogels for environmental applications.^12,13^

We selected MTMS and OTMS to provide complementary surface functions. MTMS forms a siloxane anchoring network, whereas the C18 chain of OTMS lowers surface energy. This study evaluates the response of a dual-silane MTMS:OTMS ratio series and the performance of aerogels prepared from Bleached MFC and Brown MFC; the Supporting Information discusses the mechanistic basis for the combined treatment.

This study presents an innovative dual-silane modification strategy combining methyltrimethoxysilane (MTMS) and octadecyltrimethoxysilane (OTMS). MTMS provides efficient anchoring and structural stability through a dense siloxane network, while OTMS, with its long octadecyl chain, drastically reduces surface energy. This synergistic combination creates multi-scale hierarchical roughness, enabling superior Cassie–Baxter state stability, exceptional water repellency (contact angles 134–135°), and, critically, extremely low water absorption (0.47 g g^–1^ for brown MFC). Using brown MFC, which retains residual lignin, further enhances sustainability by eliminating energy-intensive bleaching processes. The specific objectives of this study are (1) to develop and characterize hydrophobic MFC aerogels modified with the MTMS/OTMS dual-silane approach; (2) to compare the performance of bleached versus brown MFC; (3) to elucidate the mechanistic basis for the synergistic effect of dual-silane modification; and (4) to validate the practical applicability of these aerogels for oil spill remediation in marine environments.

## Results and Discussion

### Composition

Table 1 presents the average structural polysaccharide content in the leached and brown microfibrillated cellulose pulp samples, along with other physical parameters. The lignin content in the lower Bleached MFC is consistent with the bleaching procedure used, which specifically aims to remove lignin from the samples.

**Table 1:** Physical and Chemical Characterization of MFC Pulps

sample	density (g/mL)	moisture (%)	cellulose (%)	hemicellulose (%)	lignin (%)	ash (%)
bleached MFC	1.020 ± 0.017	96.2000 ± 0.0013	47.30 ± 4.18	18.15 ± 2.31	0.70 ±0.05	0.030 ± 0.019
brown MFC	1.020 ± 0.015	96.3000 ± 0.0010	43.20 ± 2.16	20.50 ± 2.69	9.70 ± 2.10	2.20 ± 0.62

### Production of Bleached and Brown MFC Aerogel Samples

Samples of aerogels produced from bleached microfibrillated cellulose (MFC) and brown MFC (measuring 47.0 ± 3.9 mm in diameter and 39.6 ± 4.8 mm in height) are shown in Figure 1, which also presents the results of tests conducted before (left) and after (right) exposure to various types of oils and water.

**Figure 1:**
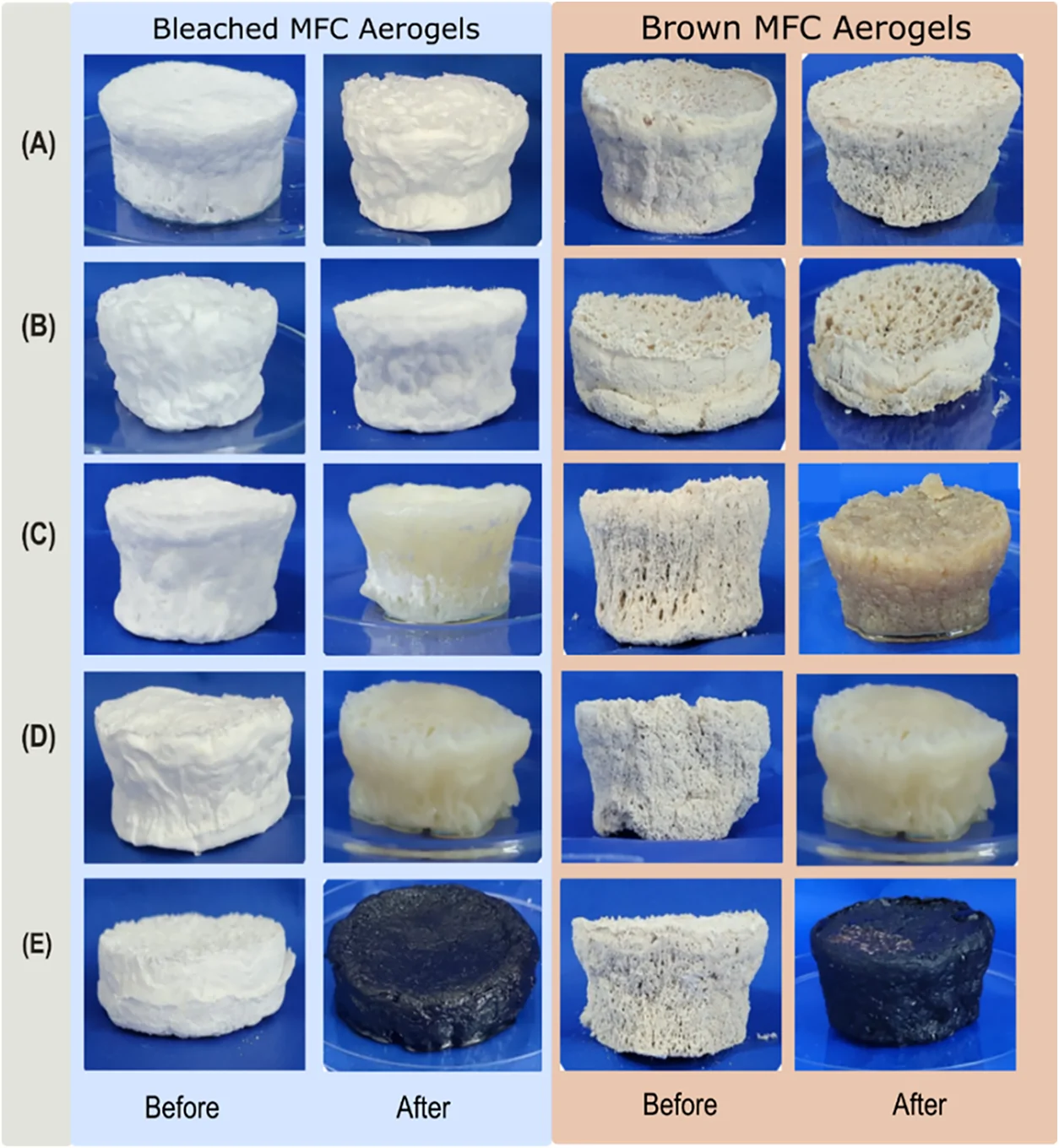
Samples of bleached MFC-based aerogels and brown MFC-based aerogels functionalized with MTMS/OTMS before and after absorption tests with Osmosis water (A), seawater (B), cooking oil (C), diesel oil (D), and petroleum (E).

The absorption capacity of both bleached MFC and brown MFC aerogels was thoroughly examined with various fluids relevant to environmental cleanup. The fluids tested included reverse osmosis water, seawater, cooking oil, diesel oil, and petroleum. Figure 1 shows the visual characteristics of these different test fluids. We determined the absorption capacity for each fluid using the method outlined in ASTM F726-17, Standard Test Method for Sorbent Performance of Adsorbents, as described in the Materials and Methodssection.

### Water and Oil Absorption Capacity of Aerogels

The production process of bleaching MFC requires a high water demand, leading to high liquid effluent, solid waste, and atmospheric emissions. Although Brown MFC avoids bleaching, this experimental study did not include a quantitative comparison of energy demand, production cost, and life-cycle impacts. Oil absorption capacity (g g^–1^) determines the hydrophobic aerogels’ absorption grade.

The superior performance of brown MFC aerogels, despite lower absolute oil absorption capacity compared to bleached MFC, is mechanistically attributed to residual lignin content. Brown MFC contains 9.70 ± 2.19% lignin (Table 1), compared to only 0.70 ± 0.05% in bleached MFC. This residual lignin creates intrinsically hydrophobic domains and reduces hydroxyl group density by approximately 2.7-fold, resulting in exceptional oil/water selectivity (36.4:1 for brown MFC vs. 3.9:1 for bleached MFC). Supporting Information Section S1 provides a comprehensive mechanistic analysis of how residual lignin reduces water absorption while maintaining oil-absorption capacity.

The absorbent Sodium alginate/sodium carboxymethyl cellulose aerogel showed an absorption capacity of 6–12 g g^–1^.^14^ An oxidized-cellulose nanofibrillated aerogel was obtained with a higher absorption capacity of 11.7–28.1 g g^–1^.^15^ Wood residues were used to produce an aerogel treated with polydimethylsiloxane to produce hydrophobic surfaces, showing a high oil absorption capacity of about 20 g g^–1^. 16 Wood aerogels were also modified with methyltrimethoxysilane, and a superhydrophobic material was obtained with an absorption capacity of 12.3–25.1 g g^–1^.^16^ Cotton stalk powder produced hydrophobic aerogel based on cellulose nanofibers-chitosan-zinc with a high absorption capacity of 15.4–47.1 g g^–1^.^17^ Cellulose nanocrystals with poly­(vinyl alcohol) (PVA), and thermal chemical vapor deposition of methyltrichlorosilane on the surface generated a highly porous, hydrophobic, and compressible aerogel with an absorption capacity of 21.2–32.7 g g^–1^.^18^ Hydrophobic magnetic hydroxyethyl cellulose-lignin aerogel was prepared with a high absorption capacity of 26.4 g g^–1^ for petroleum and 32.1 g g^–1^ for liquid paraffin.^19^ A highly mesoporous and compressible sugarcane aerogel was prepared and coated with methyltrimethoxysilane, and the hydrophobic aerogel absorbed a range of organic solvents and oils of 22.8–40.2 g g^–1^.^20^ Nanocellulose aerogel covered with polydimethylsiloxane resulted in an elastic superhydrophobic material with an absorption capacity of 24–48 g g^–1^.^21^

In this work, the aerogel absorption capacities using bleached MFC were 5.2 g g^–1^ for water, 0.48 g g^–1^ for seawater, 20.2 g g^–1^ for cooking oil, 15.6 g g^–1^ for diesel oil, and 13.3 g g^–1^ for petroleum. The absorption capacities of aerogel using brown MFC are 0.48 g g^–1^ for water, 0.6 g g^–1^ for seawater, 17.1 g g^–1^ for cooking oil, 14.9 g g^–1^ for diesel, and 12.4 g g^–1^ for petroleum. The oil absorption capacities observed for the produced bleached-MCF and brown-MCF aerogels in this work were 13.3–20.2 g g^–1^ and 12.4–17.1 g g^–1^, respectively, and fell within the high absorption indices reported in the literature.

Both aerogels passed the test simulating real-life use in oil-contaminated marine waters. Figure 2 illustrates the seawater/oil mixture absorption test using bleached MFC aerogel: (A) a sample of seawater contaminated with crude oil, (B) the result after the absorption test showing how this water looked, (C) the bleached MFC samples before and after undergoing the absorption test.

**Figure 2:**
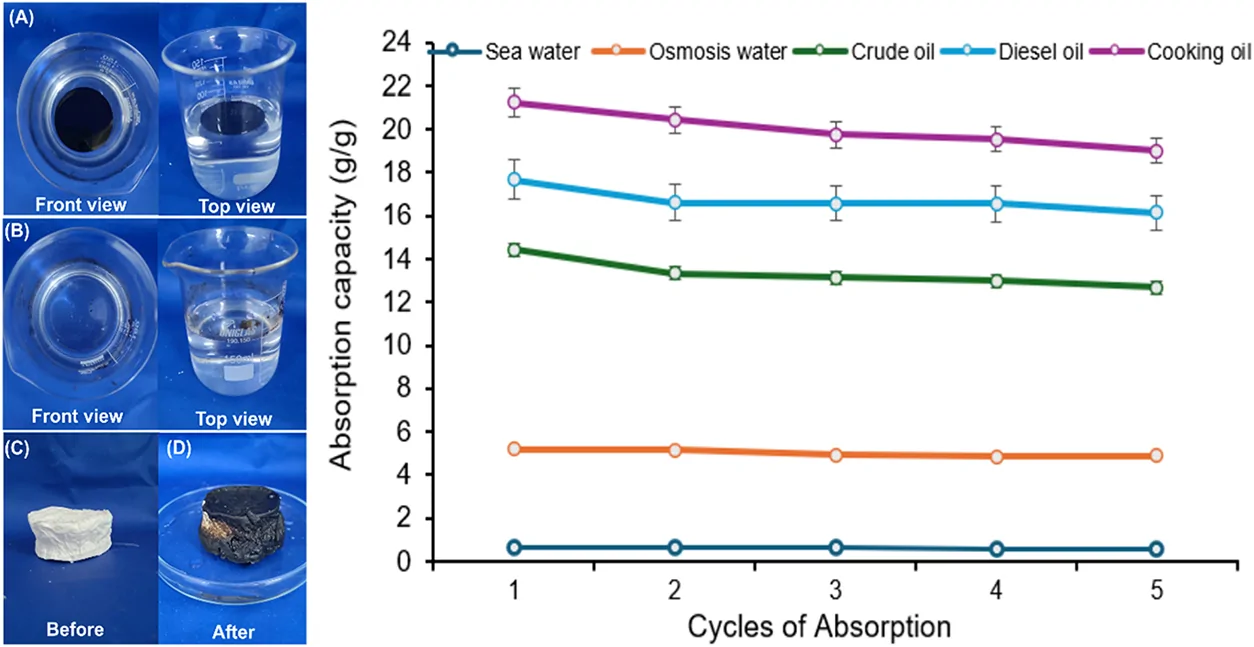
Seawater plus oil absorption test with bleached MFC aerogel. A – contaminated water before absorption test, B – seawater after absorption test, C – aerogel using bleached MFC before, and D – after absorption test.

Similar results are observed with the brown MFC aerogels, as exhibited in Figure 3. In (A) shows a sample of seawater contaminated with crude oil; (B) the cellulose submerged during the absorption test covered by oil; (C) the result after the absorption test of how this water looked; (D) the Brown MFC sample before undergoing the absorption test, and (E) after undergoing the absorption test. However, for osmotic and seawater absorption, uptake was minimal, indicating higher selectivity for oil. This selective absorption behavior suggests that the material preferentially removes oil from oil–water mixtures, resulting in a cleaner aqueous phase.

**Figure 3:**
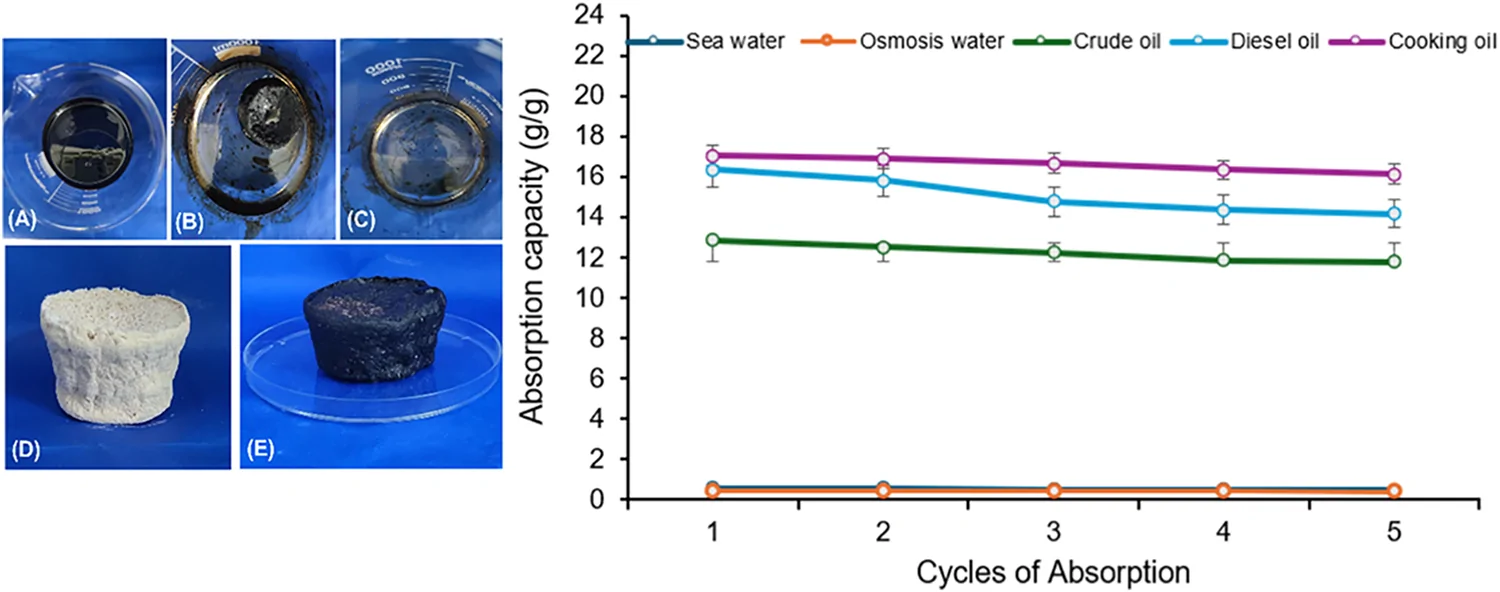
Seawater plus oil absorption test with brown MFC aerogel. A – contaminated water before absorption test, B – during a test, C – seawater after absorption test, D and E – aerogel using brown MFC before and after absorption test.

The modified aerogels were tested for reusability over five reuse cycles. The tests included seawater mixtures and reverse osmosis water. Both the prepared brown MFC aerogels and the bleached MFC aerogel showed stable water-absorption capacity. They also maintained their hydrophobic performance throughout these cycles.

However, oil-absorption capacities showed slight differences in durability and overall performance. The bleached MFC aerogel showed reduced oil absorption capacity after five reuse cycles, with declines of 12.1% for crude oil, 8.7% for diesel oil, and 10.6% for cooking oil. In contrast, the brown MFC aerogel showed a more gradual decline, with reductions of 8.50% for crude oil, 13.2% for diesel oil, and 5.4% for cooking oil. Overall, the bleached (delignified) MFC aerogel showed better maximum absorptivity values for all three tested oils: cooking oil, diesel oil, and crude oil. However, the absorption capacity order was the same for both aerogel types. The brown MFC aerogel showed similar absorption traits, but its maximum absorptivity values were consistently lower than those of the delignified version. Additionally, the solids content clearly affected the final oil absorption capacity of this aerogel system. The high hydrophobicity of the aerogel (contact angle of 134–135°) was the dominant factor, ensuring low saltwater absorption, despite small variations in ionic composition or solids content between samples, confirming the robustness of the material for real marine applications.

A superhydrophobic bacterial nanocellulose-based aerogel (s-BPGA) was prepared by Cao et al.^22^ It had a high absorption capacity (203.30 g g^–1^) and was used for 20 cycles, with absorption and desorption steps in *n*-hexane; it showed a slight decrease from the first to the second cycle, then maintained the same level through the last 20 reuses.

A hydrophobic and anisotropic aerogel was obtained with poly­(vinyl alcohol) and cellulose nanofibrils in the presence of hydrolyzed methyltrimethoxysilane sol to absorb oils and organic solvents. The authors reported good performance and reusability, with an absorption capacity above 84% after 35 absorption–desorption cycles using gasoline, resulting in less than a 16% reduction in absorption capacity.^23^

Cellulose nanocomposite aerogels were prepared with MTMS to modify the surface and prepare a superhydrophobic aerogel. The reusability of the silanized aerogel, based on its dodecane absorption capacity, showed a very high recovery rate, even after ten oil absorption/desorption cycles, with a maximum absorption capacity of 102 g g^–1^.^24^

Cellulose/graphene aerogel composites were prepared with different oxide graphene weights, and the best composition had 0.5 wt % of cellulose nanofibrillated and 20 wt % of oxide graphene to obtain a three-dimensional (3D) anisotropic absorption system and a regular geometry. Two oil desorption processes are distillation and mechanical squeezing. After 10 absorption-desorption cycles, distillation removal resulted in less than a 3% decrease in absorption capacity. With mechanical squeezing-assisted oil removal, around 15% of the oil remained in the aerogel after each cycle, resulting in a lower oil removal rate than distillation but with lower energy demand. After 10 reuse cycles, the composite retained more than 90 % of its initial mechanical squeezing absorption capacity (less than a 10% decrease).^25^

Synergistic optimization of surface roughness through dual-silane modification is critical to achieving the reported performance. MTMS provides a robust, highly cross-linked siloxane network (micro-scale roughness, 1–10 nm) that anchors securely to the cellulose substrate, while OTMS, with its long octadecyl chains, projects outward to create nano-scale roughness (2–5 nm) and drastically reduces surface energy. This hierarchical multi-scale structure cannot be achieved with either silane alone. The optimal ratio of 3:1 (MTMS:OTMS) was determined to maximize water contact angle (133.7° ± 1.2°) while maintaining structural integrity and durability. Supporting Information Section S2 provides a detailed analysis of the MTMS:OTMS ratio optimization and the synergistic mechanisms. Within the tested MTMS: OTMS series, the 3:1 formulation provided the highest contact angle (134–135°), the lowest water sorption (0.47 ± 0.08 g g^–1^), and the highest oil/water selectivity (36.4:1), while retaining oil sorption of 17.06 ± 0.80 g g^–1^ and compressive strength of 98 ± 3 kPa (Table S2.1). We therefore selected this composition as the best overall balance of water repellency, selectivity, and structural integrity.

The higher MTMS fraction favors siloxane network formation, whereas OTMS contributes low-surface-energy C18 chains; their 3:1 combination provides the hierarchical surface rationale discussed in Supporting Information Section S2.

### FTIR Analysis

Figure 4 shows the FTIR spectra of bleached MFC pulp samples and their corresponding aerogels functionalized with MTMS and OTMS, as well as brown MFC pulp samples and aerogels obtained under the same functionalization conditions using MTMS and OTMS. The overlaid presentation enables direct comparison of the spectral features of the raw and dual-silane-modified samples on a common wavenumber scale. FTIR analysis was performed to verify the successful incorporation of MTMS and OTMS.

**Figure 4:**
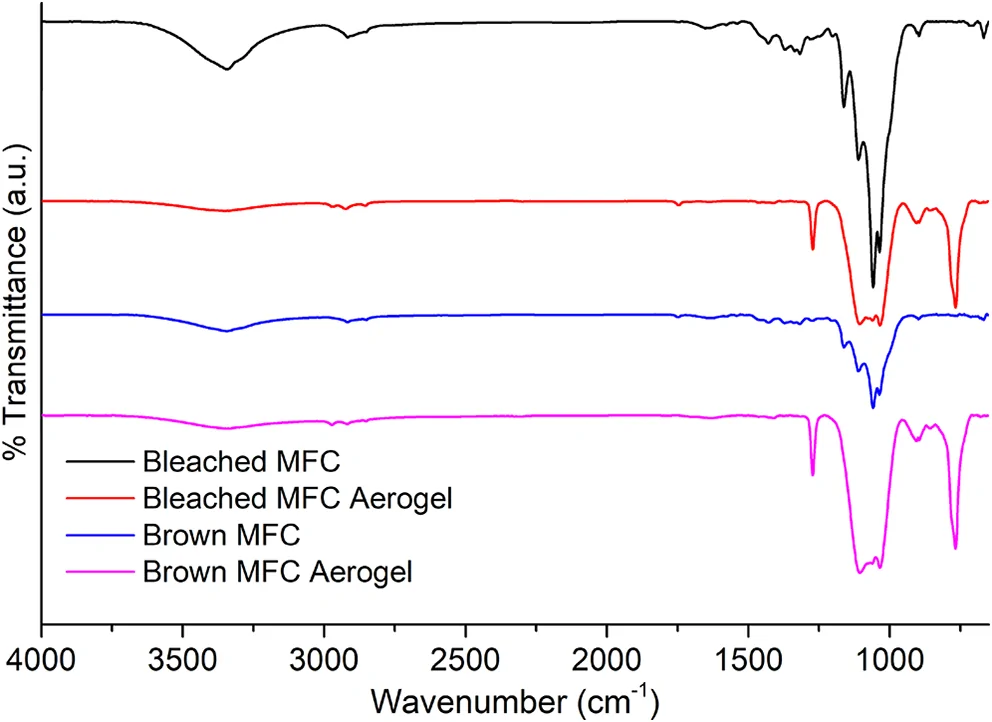
Overlaid FTIR spectra of bleached MFC pulp, brown MFC pulp, and their corresponding MTMS/OTMS-modified aerogel.

Key indicators of successful silanization include strong bands at 765–770 cm^–1^ (characteristic of CH_3_ vibrations in silane) and 1274–1275 cm^–1^ (attributed to Si-CH_3_ bonds). Furthermore, distinctive peaks between 1055 and 1311 cm^–1^, resulting from the overlap between cellulose C–O bonds and siloxane Si–O–Si bonds, support the successful incorporation of silane agents into the MFC aerogels.^26^

### Analysis of Hierarchical Structure via Scanning Electron Microscopy (SEM)

Scanning electron microscopy (SEM) was used to examine the surface morphology of both bleached and brown modified microfibrillated cellulose (MFC) samples (see Figure 5). MTMS/OTMS treatment formed silane clusters with a unique structure on the MFC surface, significantly increasing roughness. The aerogels made from these treatments showed a fairly even distribution of voids on their surfaces. These voids likely resulted from the chemical interactions between the silane coupling agents and the hydroxyl groups in the MFC matrix.^27^

**Figure 5:**
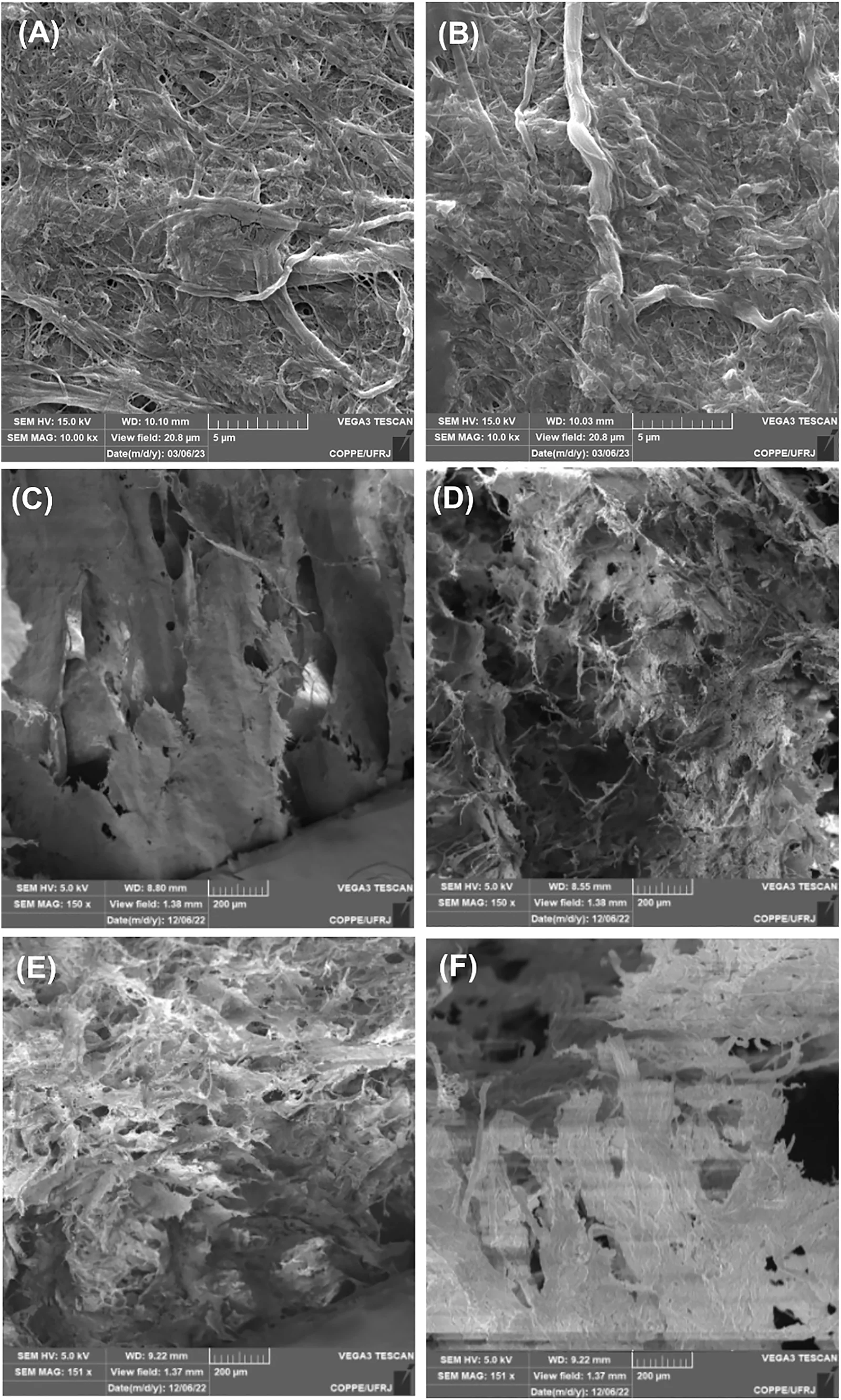
SEM micrographs of bleached and brown MFC aerogels at 5,000× magnification (overlaid for direct comparison): (A) bleached MFC; (B) brown MFC; (C) non-silanized bleached MFC aerogel; (D) dual-silanized bleached MFC aerogel; (E) non-silanized brown MFC aerogel; (F) dual-silanized brown MFC aerogel. The brown MFC aerogel exhibits slightly thicker fibers due to residual lignin content and more pronounced surface roughness than the bleached MFC aerogel. Scale bar: 200 nm.

### Thermogravimetric Analysis – TGA

Thermal stability is an essential characteristic for aerogel applications. TGA curves revealed three distinct mass-loss regions, typical of cellulose-based materials. Thermogravimetric analysis (TGA) was used to compare the thermal degradation profiles of the MFC precursors and the corresponding aerogels (Figure 6). The curves show three mass-loss regions: removal of absorbed water below 150 °C, the main degradation of the MFC-based matrix between approximately 200 and 350 °C, and decomposition of carbonaceous residue near 380 °C, as detailed in Supporting Information Section S4. This staged behavior is consistent with previous reports on silane-modified cellulose and silica–cellulose aerogels.^27,28^ The aerogels demonstrated considerable thermal stability, comparable to other cellulose aerogels.^29^ The thermal stability of cellulose nanomaterials is influenced by factors such as their chemical composition and structural properties. This inherent thermal resistance is advantageous for applications like food packaging.

**Figure 6:**
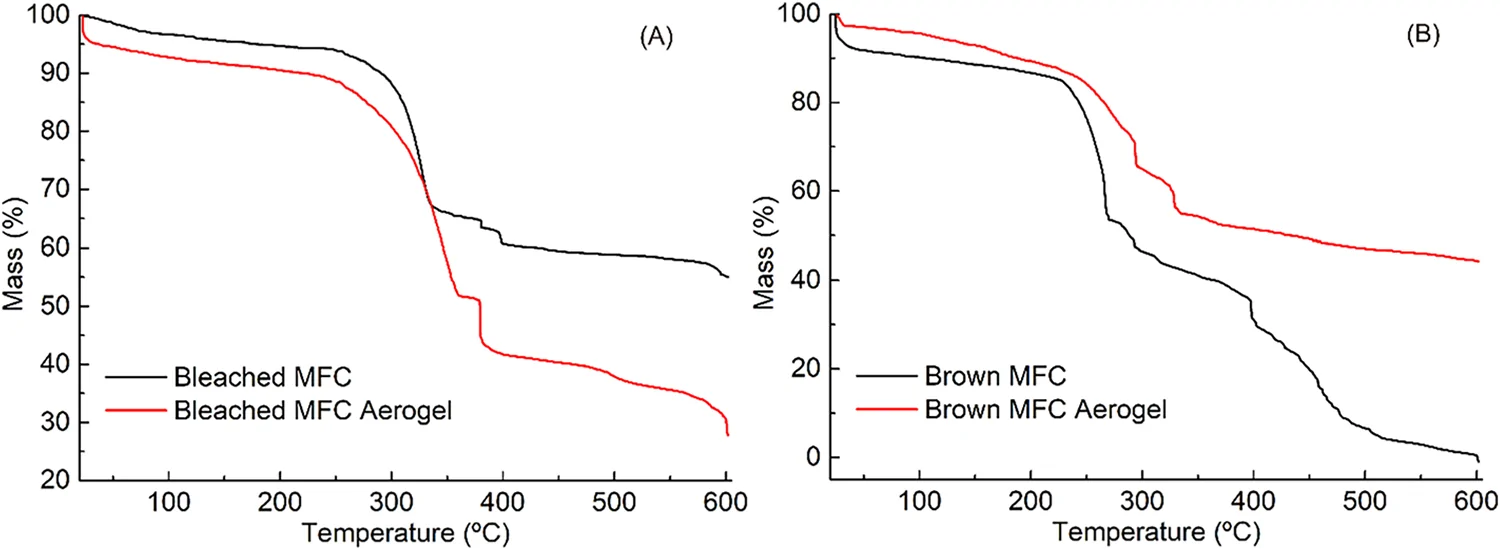
Comparative TGA curves of samples: (A) bleached MFC pulp and Bleached MFC/MTMS/OTMS Aerogel, (B) brown MFC pulp and Brown MFC/MTMS/OTMS

A significant observation in the TGA analysis is the marked shift in thermal degradation curves between bleached and brown MFC aerogels. The brown MFC/MTMS/OTMS aerogel exhibits a thermal degradation onset (*T*_onset_) approximately 40 °C higher than that of bleached MFC/MTMS/OTMS, with initial degradation beginning at ∼280 °C versus ∼240 °C. This substantial shift reflects fundamental differences in chemical composition and structural organization.

The primary factor is residual lignin, an aromatic polymer with significantly greater thermal stability than cellulose.^30^ Lignin contains 9.7 ± 2.1% in brown MFC (Table 1), compared with only 0.70 ± 0.05% in bleached MFC, a 14.4-fold difference.^31^ Due to its extensive aromatic ring structures and C–C bonds, lignin requires a higher activation energy to break than the C–O bonds in cellulose.^30,32^ Additionally, the silanization process contributes to thermal stability through the formation of siloxane (Si–O–Si) networks with bond dissociation energies of 450–500 kJ mol^–1^,^33^ compared to 350–400 kJ mol^–1^ for C–O bonds in cellulose,^34^ providing a protective coating shielding the underlying cellulose from thermal degradation. The synergistic combination of lignin’s intrinsic stability and the protective siloxane coating results in the observed 40 °C shift.

Figure 6 shows that the main mass-loss region of Brown MFC/MTMS/OTMS is shifted to higher temperature relative to Bleached MFC/MTMS/OTMS. This behavior is consistent with the higher onset temperature of Brown MFC/MTMS/OTMS and with the thermal contribution of the residual lignin. A detailed kinetic analysis is provided in Supporting Information Section S4.

The activation energy for thermal degradation (*E*_a_), calculated using the Kissinger method^35^ from TGA data acquired at heating rates of 5, 10, 15, and 20 °K min^–1^ (Section 2), increases by approximately 31% for brown MFC aerogel (*E*_a_ ≈ 180 ± 8 kJ mol^–1^) compared to bleached MFC (*E*_a_ ≈ 137 ± 8 kJ mol^–1^) (Supporting Information Section S4). This substantially greater energy requirement for thermal degradation initiation reflects the higher thermal stability of lignin-containing materials.^30^ The residual mass at 600 °C is 22–25% for brown MFC versus 18–20% for bleached MFC (Section S3), reflecting lignin’s greater charring propensity.^36^ Char yield from lignin is typically 40–50%, compared to only 5–10% from cellulose,^36^ explaining the higher residual mass in brown MFC aerogels. Supporting Information Section S3 provides a comprehensive kinetic analysis, including detailed thermal degradation mechanisms, activation energy calculations, the role of lignin and siloxane networks, and practical implications.

### Crystallinity Index Obtained by XRD

The Crystallinity index (CrI) is defined as the ratio of the crystalline to amorphous regions of cellulose.^37^ The crystalline cellulose percentage was calculated using the method outlined by,^38^ which involves determining the difference between the intensity of the crystalline peak (notably the (002) plane) and that of the amorphous component.

This deconvolution method mathematically separates the crystalline and amorphous components of the XRD pattern by fitting assumed peak shapes to the experimental data. The CrI is calculated as the ratio of the area under the crystalline peaks to the total area of the fitted pattern, which includes both the crystalline and amorphous parts.^39^

Figure 7 presents the X-ray diffraction (XRD) patterns, which display characteristic cellulose peaks at 2θ ≈ 15.87 and 22.22°, corresponding to the (110) and (200) crystallographic planes, respectively.^40^ Lignin in brown MFC influences the crystallinity index (CrI) by increasing the material’s amorphous content, thereby reducing the overall CrI relative to bleached or purified cellulose. Because lignin is inherently amorphous, it increases the amorphous background intensity, particularly around 2θ ≈ 16–18°, while the crystalline peaks remain centered around 2θ ≈ 22–23°. CrI was determined using both the Segal method and a peak deconvolution approach, yielding lower CrI values for the brown MFC samples. These results are consistent with the data in Table 2, which indicate a marked reduction in crystallinity for the lignin-containing pulp. Specifically, using the deconvolution method, the crystallinity index decreased by approximately 30.4% for the lignin-containing MFC compared to the bleached sample and by 45% for the brown MFC-based aerogel. As lignin lacks long-range crystalline order, it does not generate distinct diffraction peaks. Instead, its presence is inferred indirectly through changes in the diffractogram. A broad hump centered around 2θ ≈ 15–18°, corresponding to amorphous regions of cellulose and lignin, becomes more pronounced in samples with higher lignin content. An elevated baseline or widened hump in this region reflects an increased proportion of amorphous material, typically attributed to residual lignin and hemicellulose. This amorphous contribution diminishes the definition of the crystalline cellulose peaks, primarily by enhancing background scattering rather than introducing new peaks. Consequently, lignin masks or attenuates the intensity of the characteristic crystalline peaks, leading to an underestimation of CrI when present in significant amounts.

**Figure 7:**
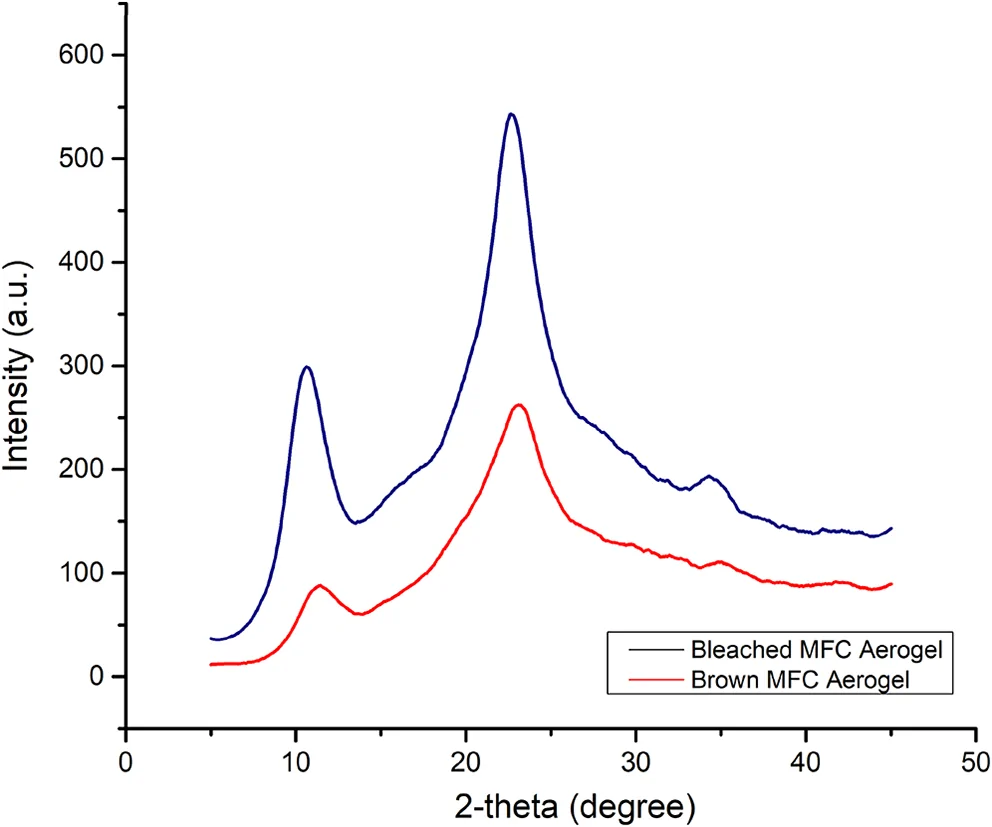
XRD patterns of raw Bleached MFC and Brown MFC pulps and of the corresponding aerogels before and after dual-silane modification.

**Table 2:** Summary of XRD Peaks for Bleached/Brown MFCs before Aerogel Production Treatment and Dual Silanized Bleached/brown MFC-Based Aerogels

	CrI (%)		main peaks (2θ)	
samples	Segal Method	deconvolution method	(110)	(200)
bleached MFC pulp	73.33	25.24	∼15.87°	∼22.22°
MTMS/OTMS/bleached MFC – aerogel	68.40	12.26	∼10.65°	∼22.71°
brown MFC pulp	72.85	17.56	∼16.1°	∼22.99°
MTMS/OTMS/brown MFC – aerogel	72.50	8.45	∼11.42°	∼23.42°

Figure 7 shows the diffraction patterns of the raw Bleached MFC and Brown MFC pulps, along with those of the corresponding aerogels, allowing direct comparison before and after aerogel preparation and silanization. The raw materials show the characteristic reflections of cellulose I summarized in Table 2, which provides the structural baseline for evaluating the modified aerogels.

The halo at 2θ ∼35° suggests the presence of at least one amorphous phase. At the same time, the distinct peaks at higher scattering angles correspond to changes during aerogel formation/silanization, as noted by Panda et al.^28^ As shown, the crystallinity indices of pulp and aerogels depend on the calculation method used. The lower crystallinity index found in aerogels made from brown MFC cellulose pulp is expected. This is because residual non-cellulosic components such as lignin and hemicelluloses are amorphous. These components disrupt the regular arrangement of cellulose chains and increase the amorphous content. Bleached pulp undergoes significant lignin removal and purification. Brown MFC pulp retains a large amount of lignin and hemicellulose. As a result, these components appear as broad, non-crystalline signals in XRD patterns.^41^

Aerogel production typically includes dispersion, solvent exchange, and drying. These stages can alter the original crystalline structure of cellulose, especially when chemical treatments such as silanization are applied. In Brown MFC pulp, these effects are more pronounced because non-cellulosic components introduce disorder, hindering the reformation or retention of crystalline regions during regeneration or drying. Consequently, lignin-containing pulps become amorphous, and processing-induced structural disorganization contributes to the low crystallinity observed in aerogels made from brown MFC.

### AFM

As discussed above and confirmed by atomic force microscopy (AFM), adding silane agents increases surface roughness. This is attributed to the reaction between silane and cellulose hydroxyl groups, which form covalent bonds that impart hydrophobicity to cellulose, a naturally hydrophilic polymer.

As shown in Figure 8, the Brown MFC surface exhibited greater average roughness (∼414 nm), than the Bleached MFC surface (∼161.8 nm), consistent with the heterogeneous lignin-containing substrate. After MTMS/OTMS treatment, the measured roughness increased to ∼581 nm for Bleached MFC and ∼482 nm for Brown MFC. This trend is consistent with reports that alkyl-silane functionalization can increase the roughness and decrease the hydrophilicity of cellulose-based materials.^42,43^ The AFM results therefore support surface functionalization, although they do not independently establish the thickness or continuity of a silane coating.

**Figure 8:**
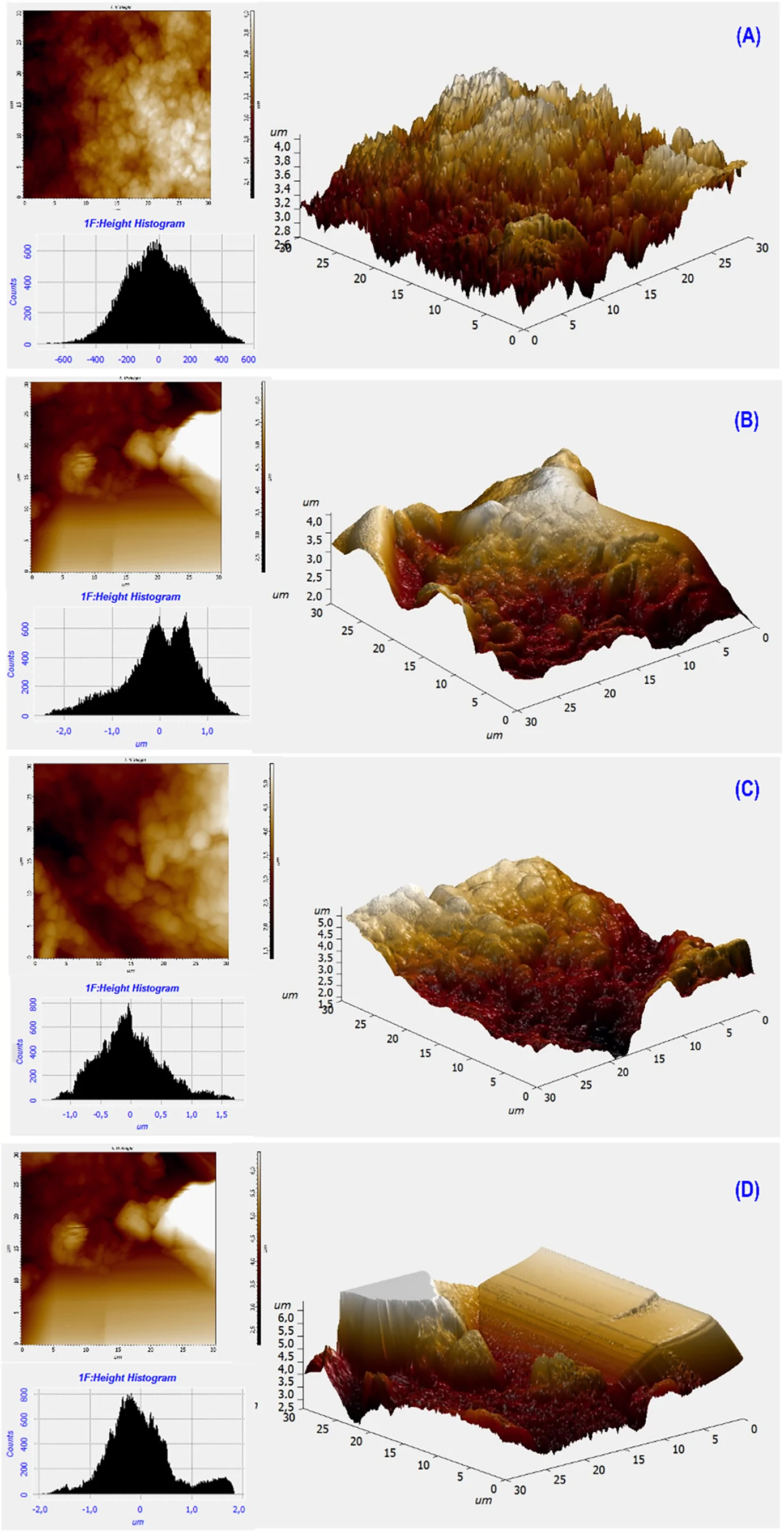
Two-dimensional and three-dimensional AFM topography images and height histograms (scan size: 30 × 30 μm) of (A) bleached MFC pulp, (B) dual-silanized Bleached MFC aerogel, (C) brown MFC pulp, and (D) dual-silanized brown MFC aerogel.

### Water Contact Angle

Materials are usually grouped by their water contact angle (WCA, θ) into superhydrophilic (θ < 5°), hydrophilic (θ < 90°), hydrophobic (90° ≤ θ ≤ 150°), and superhydrophobic (θ > 150°) and superhydrophobic (θ > 150°).^14,44,45^ Chemical modification often renders naturally hydrophilic cellulose aerogels hydrophobic, enabling selective oil absorption. WCA measurements typically quantify the resulting hydrophobicity. Literature examples include lignin/agarose/PVA aerogels (WCA ≈ 150°);^46^ mesoporous poly­(vinyl alcohol-*co*-ethylene) aerogels (WCA ≈ 147°);^47^ modified CNF aerogels (WCA ≈ 149°);^48^ bacterial cellulose/sílica aerogels (WCA ≈152°);^28^ carbonized raw cotton aerogels (WCA ≈ 131°);^49^ cellulose nanofibers-chitosan-zinc aerogels from cotton stalks (WCA ≈ 147°)^17^; carbonized/PDMS-coated nanofiber aerogels (WCA ≈ 135.8°);^50^ modified calcium alginate carboxymethyl cellulose aerogels (WCA ≈ 161.5°);^14^ MTMS-coated sugarcane aerogels (WCA ≈ 145.7°)^20^ and PDMS-coated nanocellulose aerogels (WCA ≈ 163.5°).^21^

These reported WCA values, ranging from 131 to 163.5°, span the hydrophobic and superhydrophobic regimes, highlighting the effectiveness of various modification strategies for water/oil separation applications.

The precursor microfibrillated celluloses (MFCs) used in this study were hydrophilic, with water contact angles (WCA) of 29.46° ± 0.41° for bleached MFC and 57.93° ± 3.52° for brown MFC. After MTMS/OTMS functionalization, the resulting aerogels showed significantly higher WCAs: 134.60° ± 0.40° and 133.68° ± 1.16° for bleached and brown MFC aerogels, respectively (Figure 9). Thus, these high contact angles (>90°) suggest a superhydrophobic state, which is explained by the Cassie–Baxter model.^51^ This model postulates that on a rough, hydrophobic surface, the water droplet does not penetrate the roughness valleys and remains suspended in trapped ″air pockets″. This minimizes the solid-liquid contact area, resulting in a very high apparent contact angle. These results confirm the successful surface modification, yielding hydrophobic aerogels.

**Figure 9:**
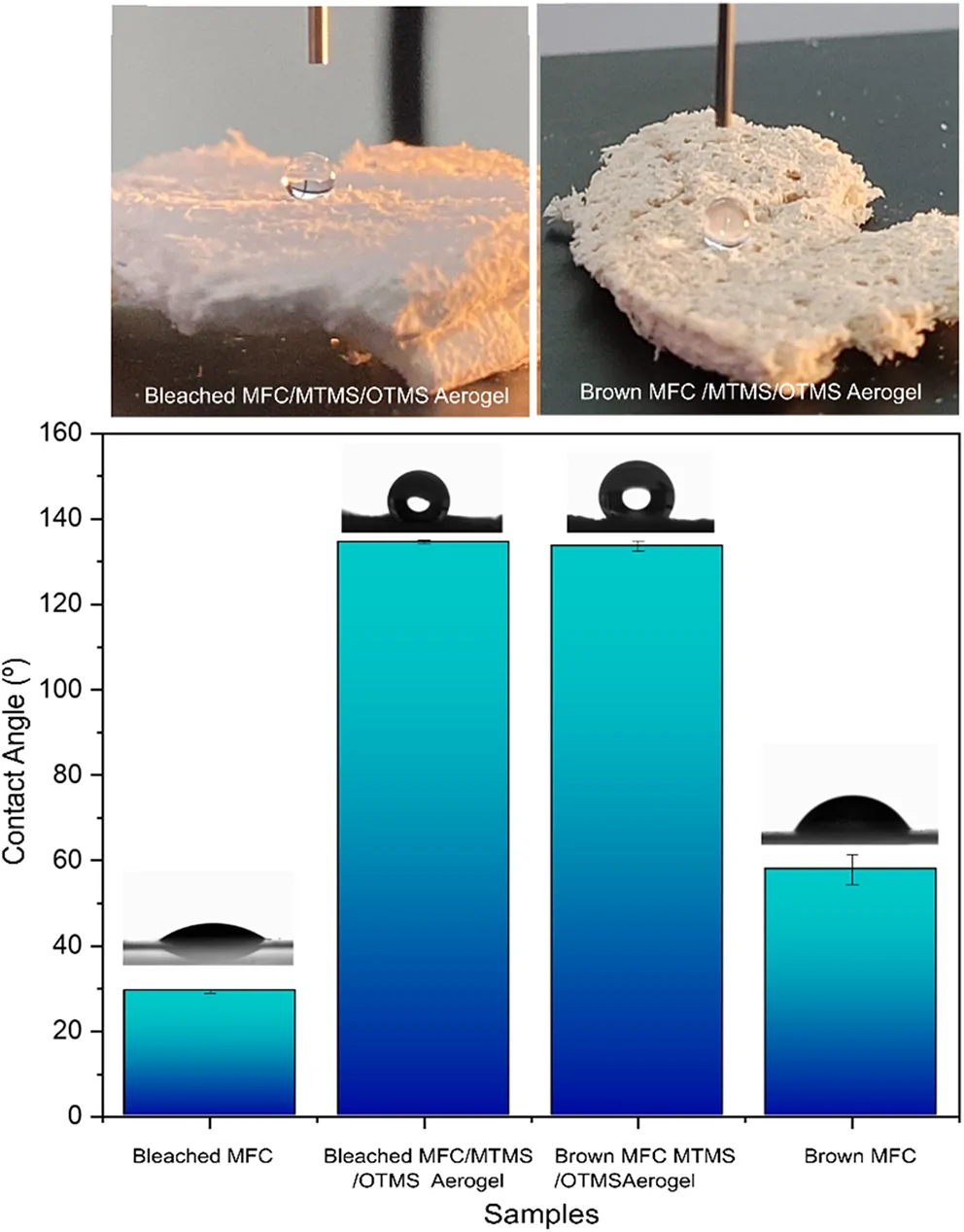
Surface contact angle analysis. On the left is a cross-section of the bleached aerogel surface, while on the right is a view of the brown MFC aerogel surface modified with silane agents. The bar graph shows contact-angle statistics (*n* = 3) for the unmodified bleached and brown aerogel surfaces and those modified with MTMS/OTMS.

### Discussion of Mechanisms of Interaction between Silanes and Cellulose

Introducing silane organic chains onto the cellulose surface is the main factor conferring hydrophobicity. The alkyl (R) chains of MTMS (methyl) and, mainly, OTMS (octadecyl, a long 18-carbon chain) are nonpolar and hydrophobic. By binding to the cellulose surface, they mask the polar hydroxyl groups and significantly reduce cellulose surface energy. In MTMS, the methyl group (−CH_3_) is relatively small but still contributes to hydrophobicity by replacing −OH groups. The three methoxy groups allow multiple bonds to cellulose and polymerization. OTMS also introduces a long 18-carbon octadecyl chain (−C_18_H_37_) that is highly hydrophobic. When these chains orient outward from the cellulose surface, they create an effective water barrier, drastically increasing the contact angle with water and, consequently, in superhydrophobicity.^52^ At the nanoscale, the formation of a silane layer on cellulose nanofibril surfaces alters topography and surface chemistry. Surface roughness at the micro and nanoscale, combined with the low surface energy of the alkyl chains, is crucial for the lotus effect and superhydrophobicity, where water droplets have minimal contact with the surface.^53^ Silane modification makes cellulose not only hydrophobic but also oleophilic (oil-loving). The same nonpolar alkyl chains that repel water attract oil molecules, which are also nonpolar.^54^

Double silanization significantly increases the hydrophobicity of the cellulose surface, as shown in the overall diagram (Figure 10). This surface modification is achieved through a two-stage process. The crucial first stage is Hydrolysis/Condensation, which permanently modifies the cellulose surface. Gadhave et al.^55^ explain the mechanism for the reaction between the silane and cellulose, as detailed in Figure 10B. This stage involves two sequential reactions: (i) Hydrolysis: Silane molecules (R-Si­(OCH_3_)_3_) react with water present in the medium to form highly reactive silanols (R-Si­(OH)_3_) and methanol. This reaction is accelerated by the medium’s acidity, (pH 3 to 4), as noted in the methodology. (ii) Condensation: The silanol groups go through two main reactions: a. They interact with one another, forming a polysiloxane network on the cellulose surface. b. They react with the hydroxyl groups (−OH) on the cellulose surface, forming strong covalent bonds (Si–O–C). This bond underpins permanent modification and increased hydrophilicity of the cellulose surface.

**Figure 10:**
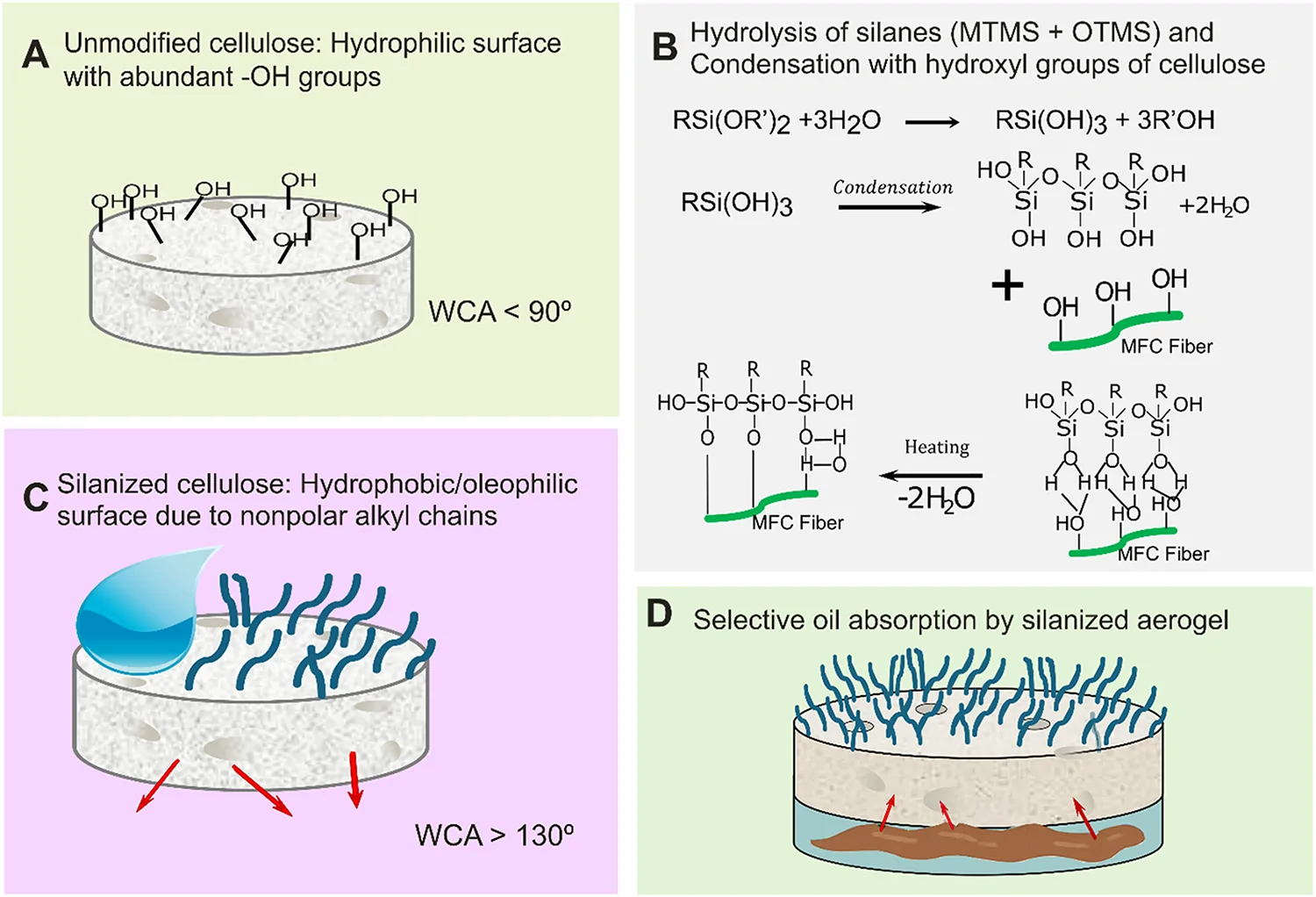
Schematic illustration of the hydrophilization mechanism of the cellulose surface via modification with double silanization: (A) the hydrophilic surface of cellulose has exposed hydroxyl groups; (B) hydrolysis reaction of MTMS and OTMS agents to form reactive silanol groups; (C) condensation reaction of silanes with the hydroxyl groups of cellulose and with each other, creating a covalently bonded polysiloxane layer; (D) the final surface structure, characterized by nanoscale roughness and oriented alkyl chains that trap air, exhibits the Cassie–Baxter superhydrophobic state, preventing water penetration and enabling selective oil absorption.

The second step consists of orienting the alkyl chains (MTMS and OTMS). Using two silanes with chains of different lengths is one of the most innovative aspects of the work. The synergistic effect arises from combining MTMS (methyl chain, −CH3) and OTMS (octadecyl chain, −C_18_H_37_), a strategic choice: MTMS, with its short chain, tends to form a more cross-linked, denser polysiloxane layer on the cellulose surface. This not only initiates hydrophobization but also contributes to nanoscale roughness. OTMS, with its long alkyl chain, orients itself outward from the surface. These long. nonpolar chains drastically reduce surface energy. The orientation of these chains, like a ″forest″ of hydrophobic molecules, prevents water penetration.

The impressive effectiveness of surface-modified cellulose aerogels as selective oil absorbents stems from the combined effects of their natural three-dimensional porous structure and tailored surface chemistry. The aerogel’s highly porous design remains intact after modification, providing a large surface area for interaction. Silanization makes the pore walls hydrophobic and oleophilic. This dual surface feature controls selective absorption. Water is repelled, while oil is attracted and quickly absorbed through capillarity into the highly interconnected pore network. The combination of high porosity and this selective affinity translate directly into exceptional oil absorption capacities.^56^

The microscale physical properties, specifically the pore size and distribution, are profoundly influenced by the original MFC structure and the freeze-drying process. These properties are key factors in absorption capacity and selectivity. The connected structure of the modified cellulose nanofibrils creates an effective capillary network, which is crucial for taking up large volumes of oil.^57^

Using both silanes, MTMS and OTMS, in the modification process provides a combined benefit in improving surface properties.^58^ MTMS, with its smaller methyl group, is thought to penetrate the cellulose structure more easily or form a dense, even base layer.

In contrast, OTMS has a long octadecyl chain that improves hydrophobicity. This makes it more noticeable and long-lasting. This dual-silanization method provides complete surface coverage and a high density of nonpolar chains, improving hydrophobicity and oleophilicity.

Cellulose modification starts with the hydrolysis of alkoxysilanes to produce silanols. These silanols then undergo condensation, forming stable covalent Si–O-cellulose bonds with the cellulose hydroxyl groups. Grafting nonpolar alkyl chains, such as methyl and octadecyl, effectively reduces the surface energy of the cellulose. This process makes the cellulose both hydrophobic and oleophilic. It operates at the nanoscale and significantly alters the surface chemistry and texture of the nanofibrils. As a result, the aerogels can selectively absorb large amounts of oil while effectively repelling water, making them very promising for large-scale environmental cleanup efforts.

Surface roughness, induced by double silanization, plays a fundamental role. The Wenzel and Cassie–Baxter models are classical models of hydrophobicity on rough surfaces.^59^ These are briefly described below.

#### The Wenzel Model (Homogeneous Wetting)

The Wenzel model describes homogeneous wetting, in which the liquid fills all the grooves and roughness on the surface. The apparent contact angle (θ_w_) is connected to the intrinsic contact angle (θ) of the flat surface and to the roughness factor (*r*), which is the ratio of the actual surface area to the projected surface area: cos θ_W_ = *r*. cos θ.

In this model, if the intrinsic surface is hydrophobic (θ > 90°), increasing the roughness (*r* > 1) amplifies the hydrophobicity, increasing the contact angle.

#### The Cassie–Baxter Model (Heterogeneous Wetting)

The Cassie–Baxter model describes heterogeneous wetting, where the liquid does not penetrate the surface grooves and remains suspended on a mixture of solid and trapped air. The apparent contact angle (θ_CB_) is given by cos θ_CB_ = *f*_s_(cos θ + 1) – 1, where *f*_s_ is the fraction of the solid surface area in contact with the liquid. In this state, trapped air (low surface energy) dominates superhydrophobicity, resulting in very high contact angles and low hysteresis.

Scanning electron microscopy (SEM) images of the unmodified aerogels provide a baseline morphological reference. Figure 5A shows the bleached MFC aerogel surface, while Figure 5B shows the brown MFC aerogel surface, both before silanization. These unmodified samples show natural fibrous structures with minimal nanoscale surface roughness.

Following dual silanization with MTMS/OTMS, the aerogel surfaces exhibit dramatically enhanced hierarchical roughness. Figure 5C shows the modified bleached MFC aerogel surface, while Figure 5D displays the modified brown MFC aerogel surface. The silanization process creates visible siloxane aggregates that significantly increase surface roughness at multiple scales, which is crucial for the Cassie–Baxter regime.

Scanning electron microscopy images (Figure 5D,5F) of the modified aerogel (with MTMOS/OTMS) provide crucial morphological evidence for determining the wetting regime. The contact angle of 134–135° and the high oil/water selectivity suggest that wetting occurs predominantly in the Cassie–Baxter regime.

The hierarchical roughness, clearly observed in the SEM images (Figure 5), allows water to adhere to the tips of the siloxane asperities, trapping air pockets in the cavities. This heterogeneous wetting state, governed by the Cassie–Baxter model, is the primary mechanism that confers hydrophobicity and low water adhesion. Although this study did not measure contact angle hysteresis, the surface morphology and high static contact angle strongly indicate that the aerogel operates in the Cassie–Baxter regime, consistent with the excellent oil/water selectivity observed.

We conducted image analysis using the Point Counting Method (Table 3), a standard stereological technique, with ImageJ software and SEM images (see Figure 5C–5F). For comparison, we also analyzed the unmodified samples (Figure 5A,B) to establish baseline morphological parameters. In this process, we overlaid a 5 × 5 grid of points on the images and counted the proportion of points falling on the solid phase (fibrous aggregates) relative to the total number of points (25). The aggregates are clusters of siloxanes, products of the silane reaction, that deposit and adhere to the surface of cellulose fibers.

**Table 3:** Quantitative Analysis of Aggregate Density of SEM Micrographs

	estimated count			
parameters	SEM C	SEM D	SEM E	SEM F
total points (*P*_T_)	25	25	25	25
points on aggregates (*P*_agreg_)	5 a 7	18 a 20	16 a 19	21
calculation of ϕ_s_ = *P*_agreg_/*P*_T_	≈6/25 = 0.24	≈19/25 = 0.76	≈19.5/25 = 0.78	≈21/25 = 0.84

According to the results in Table 3, the bleached MFC aerogel sample (Figure 5C) showed that only 24% of the apparent surface area is covered by solid material (φ_s_ ≈ 0.24). This suggests a less suitable surface for stable hydrophobicity in the Cassie–Baxter regime, potentially leading to a transition to the Wenzel regime (complete wetting of the roughness). For the silanized bleached MFC aerogel (Figure 5D): φ_s_ ≈ 0.76, indicating that approximately 76% of the apparent surface area is covered by solid aggregates. This high solid area fraction value is the morphological prerequisite that validates the effectiveness of double silanization. A high φ_s_ ensures enough solid support for the water droplet, allowing air pockets to be trapped in the remaining small valleys, stabilizing the Cassie–Baxter regime, and promoting high superhydrophobic performance.

Figure 5E shows a highly dense, intertwined fibrous structure, characteristic of a surface with high intrinsic roughness and low apparent porosity in the 2D plane. This morphology is ideal for stabilizing the air pockets beneath the droplet. This value, like that estimated for Figure 5D and much higher than that estimated for Figure 5C (φ_s_ ≈ 0.24), confirms that double silanization is highly effective. For the dual-silanized brown MFC aerogel (Figure 5F), the obtained value of φ_s_ = 0.84 indicates that 84% of the apparent surface area is composed of microasperities (fibrous aggregates). This high solid-area fraction is crucial. It ensures that when a water droplet touches the surface, most of its contact is with the hydrophobic solid material and the air pockets trapped in the small valleys. This configuration maximizes water repulsion and minimizes solid-liquid contact, achieving the essence of the Cassie–Baxter regime and resulting in high hydrophobicity (high contact angle and low sliding angle). The φ_s_ metric is therefore quantitative proof that dual silanization has achieved the desired morphological state.

These aggregates provide the micro-roughness of the hierarchical structure, which is crucial for air trapping and maintaining the Cassie–Baxter state. This roughness at multiple scales (micro- and nanoscales) is ideal for air trapping, as shown in Figure 8.

The stability of the Cassie–Baxter regime is critically dependent on the solid fraction (φ_s_) of the underlying fibrous structure.^30^ For our brown MFC aerogel with φ_s_ = 0.84 (determined by point counting method on SEM images, as described in the Section 2), the Cassie–Baxter equation (cos θ_CB_ = φ_s_ cos θ_0_ – (1 – φ_s_)), with effective Young contact angle θ_0_^eff^ = 128.4°, predicts a theoretical contact angle of 133.0°. This prediction matches our experimental observation of 133.68° ± 1.16° (Table 3), validating stable Cassie–Baxter operation. The excellent agreement between theoretical prediction and experimental observation (difference of only 0.68°) confirms that our aerogels operate in the Cassie–Baxter regime rather than the Wenzel regime.^31,32^

This stability is physically grounded in the intertwined fibrous structure: the hierarchical organization of fibers (macro-scale, 50–200 nm diameter) (this work),^60^ silane-derived aggregates (micro-scale, 1–10 nm),^61^ and extended alkyl chains (nano-scale, 2–5 nm)^61^ create an ideal interface for water droplet suspension.^30^ The solid fraction of 0.84 means 84% of the apparent surface area is solid material, while 16% consists of air-filled valleys—the optimal balance for Cassie–Baxter stability.^30^ This geometry provides enough solid support for water droplets while maintaining sufficient void space to trap air pockets. In contrast, non-silanized bleached MFC aerogel, with φ_s_ = 0.24 (Section S4), lacks sufficient solid support, transitioning to Wenzel wetting with reduced contact angles (60–70°) and increased water absorption (5.20 ± 0.42 g g^–1^, Table 1).

The robust fibrous structure mechanically supports air pockets and prevents their collapse even under compression, maintaining Cassie–Baxter stability during repeated absorption/desorption cycles. Our experiments show that after 5 cycles of oil absorption and desorption, the contact angle remains above 131° (Section S4), with only 6.8% reduction in oil absorption capacity. This temporal stability indicates that the Cassie–Baxter regime remains stable over practical time scales. The exceptional durability is attributed to the robustness of the siloxane network,^62,63^ which does not degrade significantly during repeated cycling, and the mechanical resilience of the fibrous structure.^63^ A detailed analysis of Cassie–Baxter regime stability in relation to fibrous structure, including quantitative relationships between solid fraction (φ_s_) and contact angle, mechanical perturbation resistance, and temporal stability over repeated cycles, is provided in Supporting Information Section S4. This analysis validates the Cassie–Baxter model with excellent agreement between theoretical predictions and experimental observations (difference of 0.68°).

## Materials and Methods

### Materials and Reagents

Bleached microfibrillated cellulose pulp (Bleached MFC) and unbleached microfibrillated cellulose, denominated as Brown MFC, were kindly donated by Klabin S/A and processed as received. The silanizing agents Octadecyltrimethoxysilane (OTMS – technical grade) and Trimethoxymethylsilane (MTMS -purity 95%) were obtained from Sigma-Aldrich. All reagents were utilized as obtained, without further purification. All reagents were analytical grade and used immediately in the study without further purification.

The samples were prepared using 250 mL glass and plastic beakers, a 100 mL graduated cylinder, a goldfish, a 2 mL pipette, pH strips, a magnetic stirrer (IKA RH Basic 1), an analytical balance, an oven and a freezing dryer.

### Characterization of Microfibrillated Cellulose Pulps

Bleached and brown MFC, also known as brown microfibrillated cellulose, were kindly provided by Klabin S.A. The characterization of these pulps in terms of cellulose, hemicellulose, lignin, and ash content was conducted following the protocols established by Ververis et al.^34^ and Sluiter et al.^64^ Briefly, the samples were first measured for density and moisture content. They were then freeze-dried to preserve the microfibrillated cellulose structure and ensure complete water removal. This procedure was performed in triplicate. Then, 0.3 g of the dry MFC was mixed with 3.0 mL of 72% sulfuric acid and incubated at 30 °C for 1 h. After incubation, water was added to create a 4% acid dilution, and the mixture was autoclaved at 121 °C for 1 h. The hydrolysates were then filtered into crucibles previously calcined at 575 °C for 5 h. The crucibles were dried in a muffle furnace at 100 °C for 24 h to facilitate later calculation of lignin percentage. After drying, the crucibles were weighed and returned to the muffle furnace at 550 °C for 5 h to determine the ash percentage.

### Preparation of Aerogels

Both types of MFC were diluted in osmosis water at a ratio of 13.8 g of MFC per 100 mL of solution. A suspension was created using the magnetic stirrer. The pH was then adjusted by adding 3 M hydrochloric acid until the medium reached a pH of 3 to 4. Under stirring, 2 mL of OTMS was added to the mixture, followed by 1.1 mL of MTMS. The suspension was stirred for another hour at room temperature. The MFC solution containing the agents was kept in the muffle furnace at 70 °C for 2 h. Silane agents require changes in pH, agitation, and temperature to bind to the MFC fibers, promoting cellulose hydrophobization.^65^

The prepared solution was frozen at −80 °C in a freezer (Thermo Scientific Forma 900 series) for 24 h before lyophilization in a lyophilizer (Terroni Científicos – LS6000) with a pump (Platinum DV-285N-250 10CFM) until complete sublimation. Figure 11 presents a flowchart summarizing the procedures described above.

**Figure 11:**
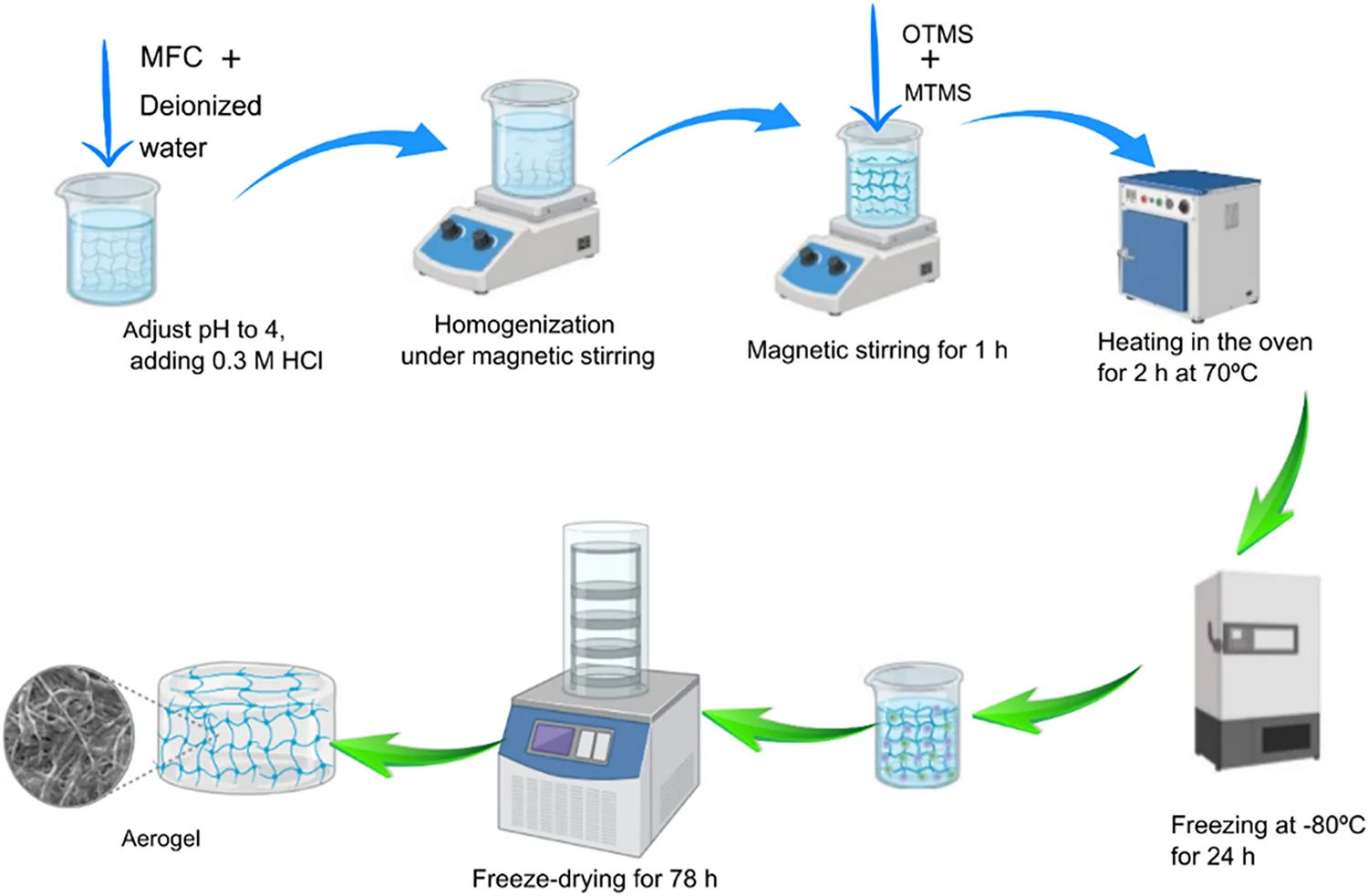
Production of hydrophobic aerogel based on MFC.

### Characterization of Aerogels

#### Water and Oil Absorption Capacity of Aerogel

We weighed the aerogels and placed them in vials containing the analysis liquids: osmosis water and seawater. We evaluated water absorption capacity using seawater samples from different locations (Arraial do Cabo and Guanabara Bay). Although specific salinity was not determined, the solids content was measured in 33.400 mg L^–1^ to characterize the aqueous medium. The experiment used several oil types, including cooking oil, diesel (S10 type), and crude oil. This variety was selected to provide comprehensive data on the biomaterial’s maximum saturation capacity for all liquid types.

The aerogel was immersed in the analysis liquid for 15 min. After this period, the samples were drained for 30 seconds using tweezers and then weighed again. Following this, the aerogels were returned to the vial for 45 min to complete a 1 h immersion analysis. The same drainage and weighing procedures were repeated after this immersion. The method followed ASTM F726-17, Standard Test Method for sorbent performance of adsorbents for use on crude oil and related spills.^66^

The tests used a mixture of seawater and oil to simulate a real-world scenario involving the developed biomaterial. This method accurately represents oil-spill contamination of seawater, enabling us to evaluate the aerogel’s effectiveness in thoroughly decontaminating seawater. We selected various oils to provide comprehensive information on the biomaterial’s maximum saturation capacity across different liquid types. The aerogel remained in the analysis for 15 min, after which we drained the samples for 30 seconds using tweezers and weighed them again. Afterward, we returned the aerogels to the vial for an additional 45 min to complete the 1-h immersion analysis, then repeated the drainage and weighing procedure. The same tests were carried out using a mixture of seawater and oil to evaluate a realistic application scenario, simulating seawater contaminated by an oil spill and the use of aerogel to decontaminate seawater.

### Fourier Transform InfraRed Spectroscopy

Fourier transform infrared spectroscopy analyses were performed using FTIR equipment (Frontier FT IR model – serial number 113646, PerkinsElmer). Each spectrum was obtained from 32 scans, with wavenumbers between 650 and 4000 cm^–1^, using attenuated total reflection (ATR) with germanium (Ge).

### SEM Measurements

The surface morphological characterizations of the MFC-aerogel were observed using a scanning electron microscope, SEM Tescan (model Veja 3). The samples were coated with a thin layer of gold by vacuum sputtering (20 mA for 2 min) to make them conductive, and were observed at 15 kV, with the fields of interest documented in micrographs.

### Determination of Solid Fraction (φ_s_) for Cassie–Baxter Analysis

The solid fraction (φ_s_), defined as the fraction of the surface occupied by solid material (as opposed to air), was determined through quantitative image analysis of SEM micrographs acquired at 5000× magnification. This parameter is critical for validating the Cassie–Baxter wetting model^51^ and understanding the stability of the superhydrophobic regime.^67^ Image analysis was performed using ImageJ software with the Point Counting Method, where a 5 × 5 grid of points was overlaid on each image, and the proportion of points falling on solid material was counted. A minimum of 10 different regions were analyzed for each sample to obtain representative average values. Aerogel samples were fractured under liquid nitrogen to obtain fresh cross sections and mounted on SEM stubs using carbon tape. Samples were sputter-coated with gold (5 nm thickness) to improve conductivity and image quality.

### Thermogravimetric Analyses

Thermogravimetric analyses (TGA) were conducted at a heating rate of 10 °C min^–1^, from room temperature (approximately 25 °C) to 600 °C. The measurements were performed in an unsealed platinum crucible under a nitrogen atmosphere at a flow rate of 50 mL min^–1^. The equipment used for these analyses was a Shimadzu model DGA-60. Samples ranged from 1 to 3 mg. This method determined the temperature at which thermal degradation starts and the mass change as temperature increased.

#### Thermogravimetric Analysis (TGA) – Kissinger Method

Thermal stability and degradation kinetics of the aerogels were evaluated using thermogravimetric analysis (TGA) conducted with a heating rate program designed for kinetic analysis via the Kissinger method. Aerogel samples (∼1–5 mg) were placed in open aluminum pans and dried at 105 °C for 2 h prior to analysis to remove residual moisture.

##### Experimental Parameters

Room temperature (25 °C) to 800 °C. Five heating rates were used for kinetic analysis: 5, 10, 15, 20, and 25 K min^–1^. Nitrogen gas at 50 mL min^–1^ (inert atmosphere to prevent oxidation). 1–5 mg per measurement.

##### For Kissinger Method for Activation Energy Calculation

The Kissinger method^35^ was employed to calculate the apparent activation energy (*E*_a_) from the temperature dependence of the degradation rate. The method utilizes the following eq 1

1 $$\ln\left(\frac{\beta }{T_{p}^{2}}\right)=\ln\left(\frac{\mathrm{AR}}{E_{a}}\right)-\frac{E_{a}}{RT_{p}}$$

Where: β = heating rate (K min^–1^); *T*_p_ = peak temperature in the derivative thermogravimetric (DTG) curve (K); *A* = pre-exponential factor; *R* = universal gas constant (8.314 J/(mol·K)) and *E*_a_ = apparent activation energy (kJ mol^–1^). For each heating rate (5, 10, 15, 20, 25 K min^–1^), we identified the peak temperature (*T*_p_) from the derivative thermogravimetric (DTG) curve. A plot of ln­(β/*T*_p_^2^) versus 1/*T*_p_ was constructed, and the activation energy was calculated from the slope using linear regression. We reported the average energy activation and its standard deviation across the five heating rates.

Onset temperature (*T*_onset_): Temperature at which 5% mass loss occurs. Peak degradation temperature (*T*_p_): Temperature of maximum degradation rate (from DTG); Activation energy (*E*_a_): Calculated via Kissinger method. Char yield: Residual mass at 800 °C (expressed as percentage of initial mass). Using five different heating rates provides robust kinetic data and enables accurate activation-energy determination.^68^ This approach is standard in thermal kinetics studies and provides more reliable results than single-rate TGA analysis.

### XRD Measurements

X-ray diffraction analyses were performed using a Rigaku MiniFlex II Desktop X-ray Diffractometer to analyze the crystalline structure of MFC fibers with and without treatment over 2θ from 5 to 45° at 1° per min. Nano ICr 2.0 software^69^ was used to calculate the crystallinity index (CrI) for different samples using two methods.^70^ The first method was the Segal method, summarized in eq 2:

2 $$CrI\left(\%\right)={\frac{I_{200}-I_{AM}}{I_{200}}}^{*}100\%$$

where CrI = crystallinity index (%); *I*_200_ = maximum diffraction intensity corresponding to the crystalline material, *I*_AM_ = minimum diffraction intensity belonging to the amorphous region.

The second method used was deconvolution, which is based on peak area and compares the crystalline and amorphous areas by separating them using a Gaussian function, as shown in eq 3.

3 $$CrI\left(\%\right)={\frac{A_{c}}{A_{c}+A_{am}}}^{*}100\%$$

where *A*_c_ represents the total integral of the crystalline area, and *A*_C_ + *A*_am_ denotes the complete integration of all split peaks, including the amorphous one.

The analysis of crystalline aerogels derived from lignocellulose, which typically contain a combination of crystalline cellulose, amorphous cellulose, hemicellulose, and lignin, benefits significantly from deconvolution methods. Unlike the Segal method, which provides a single crystallinity index based on selected intensity values, the deconvolution method fits curves across the entire X-ray diffraction (XRD) profile using Gaussian, Lorentzian, or Voigt functions. This enables more precise separation and quantification of individual crystalline peaks and amorphous contributions.^71^

### Atomic Force Microscopy Measurements

Traditional analyses of the aerogels were performed using an AFM with a Solver Nano (NT-MDT). The analysis required meticulous sample preparation: a small number of aerogels were placed in Petri dishes and dried in an oven at 50 °C for approximately 3 h. This careful process of freezing and sublimation was crucial for achieving thicknesses in the 0.10–0.20 mm range, ensuring reliable, accurate analysis with the equipment.

### Contact Angle Measurement

An optical contact angle meter (Dataphysics, model OCA 25, Serial No: O64Eo22oF8oE) was used to measure the contact angle in accordance with ISO 27448:2009. A micro syringe (5 μL) was used to drip deionized water onto the material, and a photo of the drop was taken every 1 min, displayed on the PC for calculating the contact angle.

## Conclusion

This study presents a comprehensive investigation of hydrophobic microfibrillated cellulose aerogels synthesized via dual-silane modification. The key findings demonstrate that (1) Dual-silane modification (MTMS:OTMS = 3:1) creates hierarchical multi-scale roughness (micro-scale: 1–10 nm; nanoscale: 2–5 nm) that enables stable Cassie–Baxter wetting with water contact angles of 133.7° ± 1.2° and solid fraction φ_s_ = 0.84. (2) Brown MFC aerogels achieve exceptional oil/water selectivity (36.4:1) compared to bleached MFC (3.9:1), a 9.3-fold improvement, attributed to residual lignin (9.67% vs. 0.67%). (3) Oil absorption capacities (12.4–17.1 g g^–1^ for brown MFC; 13.3–20.2 g g^–1^ for bleached MFC) are comparable to or exceed literature values while using a more sustainable starting material. (4) Superior thermal stability is observed in brown MFC aerogels (*T*_onset_ + 40 °C; *E*_a_ +31%), mechanistically attributed to residual lignin and protective siloxane coating. (5) Durability testing over 5 cycles demonstrates only 6.8% reduction in oil absorption capacity, confirming practical applicability. (6) The use of Brown MFC avoids the bleaching treatment; however, a quantitative comparison of energy demand, production cost, and life-cycle impacts was outside the scope of this study. Dedicated techno-economic, life-cycle, and scale-up assessments are required before such advantages can be established. These findings show that dual-silane-modified brown MFC aerogels are effective, durable, and sustainable sorbents for oil spill remediation in marine environments such as Guanabara Bay.

In summary, this study successfully developed hydrophobic MFC aerogels via dual-silane modification, demonstrating that Brown MFC delivers superior performance compared to bleached alternatives while reducing environmental impact and production costs. The materials achieve exceptional oil/water selectivity (36.4:1 for brown MFC), high oil absorption capacity (17.1 ± 0.8 g g^–1^), minimal water absorption (0.50 ± 0.08 g g^–1^), enhanced thermal stability (40 °C *T*_onset shift_), and excellent durability over repeated cycles. Mechanistic analysis reveals that residual lignin, hierarchical surface roughness, and protective siloxane networks synergistically create materials ideally suited for oil spill remediation in marine environments. These findings provide both practical solutions for environmental cleanup and theoretical insights into sustainable materials design.

## Supplementary Material



## Data Availability

The data sets used in this study are available by request to the corresponding author.

## Notes

Section S1: Mechanistic analysis of residual lignin; Section S2: Hierarchical roughness optimization (MTMS:OTMS); Section S3: Thermal degradation and kinetic analysis; and Section S4: Cassie–Baxter regime stability and wetting behavior (PDF)

## Abbreviations

AFM: atomic force microscopy
CrI: crystallinity index
FTIR: Fourier transform infrared spectroscopy
MFC: microfibrillated cellulose
MTMS: methyltrimethoxysilane
OTMS: octadecyltrimethoxysilane
SEM: scanning electron microscopy
TGA: thermogravimetric analysis
WCA: water contact angle
XRD: X-ray diffraction
