# Supplementary material for: Hydrophobic Microfibrillated Cellulose Aerogels via Dual-Silane Modification for Sustainable Oil Spill Recovery
Source: Langmuir. 2026 Oct 5;42(41):30646–62. doi: 10.1021/acs.langmuir.6c03349 (PMC13689925; doi:10.1021/acs.langmuir.6c03349)
Supplement: Supplementary file 1 [file la6c03349_si_001.pdf]

Supporting Information

Hydrophobic Microfibrillated Cellulose Aerogels via Dual-Silane Modification for Sustainable Oil Spill Recovery

Ninoska Isabel Bojorge Ramirez[1,], Isabela Rosa Marchette[1], Bárbara Cristina Rodrigues[1], Eliana Mosse Alhadeff[2], Marta Cecilia Tapia Reyes[3], Ruan Stevan de Almeida Ribeiro[1], Troner Assenheimer de Souza[1], Juan Manuel Pardal[4], Felipe Silva Semaan[5]*

1. Department of Chemical and Petroleum Engineering, Fluminense Federal University. R. Passo da Pátria, 156 – sala 235 – Bloco E, São Domingos –24210-240, Niterói – RJ, Brazil
2. Department of Biochemical Engineering, Federal University of Rio de Janeiro, Rio de Janeiro, Brazil, Sala 3, Av. Horácio Macedo, 2030, Cidade Universitária - Ilha do Fundão, Rio de Janeiro, RJ, 21941-909, Brazil
3. Department of Naval Engineering, Federal University of Rio de Janeiro, Av. Athos Silveira, 149 - CT – Bl. C - Sala 207, Cidade Universitária - Ilha do Fundão – RJ, 21.945-970, Brazil
4. Department of Mechanical Engineering, Fluminense Federal University, Brazil, Rua Passo da Pátria, 156 Bl. D, Campus da Praia Vermelha, Niterói, RJ, 24210-240, Brazil
5. Department of Analytical Chemistry, Fluminense Federal University, Outeiro São João Batista, sn, Campus do Valonguinho, Centro, Niterói, Rio de Janeiro, Brazil

* Corresponding author (nbojorge@id.uff.br)

SUMARY

SECTION S1: MECHANISTIC ANALYSIS OF RESIDUAL LIGNIN 1
S1.1 Introduction 1
S1.2 Molecular Structure and Properties of Lignin 1
S1.3 Molecular Mechanism of Reduced Water Sorption 1
S1.4 Quantitative Analysis: Relationship between Lignin Content and Water Sorption 2
S1.5 Implications for Sustainability 3
S1 Reference 3
SECTION S2: HIERARCHICAL ROUGHNESS OPTIMIZATION (MTMS:OTMS) 5
S2.1 Introduction 5
S2.2 Molecular Mechanism of Synergistic Interaction 5
S2.3 Multi-Scale Roughness Formation 5
S2.4 MTMS:OTMS Ratio Optimization 6
S2.5 Structural Integrity Considerations 7
S2.6 Comparison with Literature 7
S2 Reference 7
SECTION S3 - DETAILED ANALYSIS OF THERMAL DEGRADATION AND KINETIC PARAMETERS 9
S3.1 Introduction 9
S3.2 Three-Stage Thermal Degradation Mechanism 9
S3.3 Quantitative Kinetic Analysis Using Kissinger Method 10
S3.4 Role of Residual Lignin in Thermal Stability 12
S3.5 Role of Siloxane Network in Thermal Protection 13
S3.6 Derivative Thermogravimetry (DTG) Analysis 14
S3.7 Char Yield and Residual Mass Analysis 15
S3.8 Practical Implications for Oil Spill Remediation 15
S3.9 Comparison with Literature 16
S3 Reference 16
SECTION S4 - ANALYSIS OF CASSIE-BAXTER REGIME STABILITY AND WETTING BEHAVIOR. SUPPORTING INFORMATION 18
S4.1 Introduction 18
S4.2 Theoretical Foundation: Cassie-Baxter vs. Wenzel Models 18
S4.3 Experimental Validation of Cassie-Baxter Regime 19
S4.4 Role of Intertwined Fibrous Structure in Cassie-Baxter Stability 21
S4.5 Quantitative Relationship Between Solid Fraction and Contact Angle 22
S4.6 Temporal Stability During Repeated Cycles 23
S4.7 Comparison with Literature 24
S4.8 Mechanistic Insights 24
S4 Reference 24

SECTION S1: MECHANISTIC ANALYSIS OF RESIDUAL LIGNIN

S1.1 Introduction

Residual lignin in unbleached cellulose aerogels (brown MFCs) plays a critical role in determining hydrophobicity and oil/water selectivity. This section provides a detailed mechanistic analysis of how lignin content affects water sorption properties.

S1.2 Molecular Structure and Properties of Lignin

Lignin is a three-dimensional aromatic polymer composed primarily of three phenylpropane monomers: p-coumaryl alcohol, coniferyl alcohol, and sinapyl alcohol.[1] These units are connected via ether and carbon-carbon bonds, creating an extensively cross-linked aromatic network.[2] Unlike cellulose, which contains three hydroxyl groups per glucose unit ($C_6H_{10}O_5$), lignin contains significantly fewer hydroxyl groups relative to its molecular weight, averaging approximately 0.5 -1 hydroxyl group per phenylpropane unit.[3]

The three-dimensional structure and aromatic character of lignin give it fundamentally different properties from those of linear and crystalline cellulose. Aromatic rings in lignin are thermodynamically stable and resist chemical and thermal degradation much more effectively than glycosidic linkages in cellulose.[4] This intrinsic stability is one of the reasons why lignin remains in the material during processing, while cellulose is more easily modified or degraded.

Table S1.1: Comparison of Structural Properties of Cellulose vs. Lignin

Properties	Cellulose	Lignin	Reference
Unit Molecular Formulae	$C_6H_{10}O_5$	$C_9H_{10}O_3$ (approximated)	1, 2
Hydroxyl Groups per Unit	3	0.5-1	3
Structure	Linear, crystalline	Aromatic, Amorphous	2, 4
Predominant Bonds	C-O (glycosidic)	C-C, C-O-C (ether)	1, 3
Bond Dissociation Energy (kJ/mol)	350-400	400-500	4
Hydroxyl Group Density (per nm²)	3.4-4.0	0.8-1.2	5

S1.3 Molecular Mechanism of Reduced Water Sorption

Hydrogen bonds govern interactions between water and cellulose, between water molecules and the abundant hydroxyl groups on the cellulose surface.[6] Each water

molecule can form up to 4 hydrogen bonds, and the hydroxyl groups of cellulose provide multiple donor and acceptor sites.[7]

The presence of lignin fundamentally reduces the availability of hydroxyl groups for binding with water. While pure cellulose has a hydroxyl group density of 3.4-4.0 per nm² of surface area, lignin reduces this density to 1.1-1.5 per nm², a 2.68-fold reduction.[5] This reduction has dramatic consequences for water sorption.

In Bleached MFC:[8]

Hydroxyl group density:~3.4-4.0 per nm²

- Hydrogen bonding network: Extensive
- Observed water sorption: 5.2 ± 0.4 g g^{-1}

In Brown MFC:[8]

- Hydroxyl group density: ~1.1-1.5 per nm² (2.68x reduction)
- Hydrogen bonding network: Reduced
- Observed water sorption: 0.5 ± 0.1 g g^{-1} (11x reduction)

The non-linear reduction (2.68x in hydroxyl density corresponds to 11x in water sorption) suggests that the effect of lignin on hydrophobicity is amplified by the silanization process.[9] This synergistic effect is critical to understanding why silanized brown MFC material performs so superiorly.

S1.4 Quantitative Analysis: Relationship between Lignin Content and Water Sorption

Quantitative analysis of the relationship between lignin content and water sorption reveals important patterns that underpin materials design.[10]

Table S1.2: Quantitative Relationship between Lignin and Water Sorption

Sample	Lignin (%)	OH density (per nm²)	Water sorption (g g^{-1})	Oil:Water selectivity	Reference
Bleached MFC pulp	0.67 ± 0.05	3.4-4.0	-	-	8
Brown MFC pulp	9.67 ± 2.09	1.1-1.5	-	-	8
Bleached MFC/MTMS/OTMS aerogel	0.67 ± 0.05	2.1-2.5	5.17 ± 0.42	3.9:1	8
Brown MFC/MTMS/OTMS aerogel	9.67 ± 2.09	0.8-1.2	0.47 ± 0.08	36.4:1	8

Quantitative Interpretation:

Brown MFC contains 9.67% lignin compared to only 0.67% in bleached MFC, representing a difference of 14.4 times.[8] This substantial difference in lignin content corresponds to a 2.68-fold reduction in hydroxyl group density.[5] When the double-silane modification (MTMS/OTMS) is applied, the effect is amplified: water sorption is reduced from 5.17 g g^{-1} to 0.47 g g^{-1}, representing an 11-fold reduction.[8] This non-linear reduction suggests that residual lignin not only reduces the availability of hydroxyl groups, but also interacts synergistically with silanizing agents to create a surface with an even more pronounced hydrophobic character.

Oil/water selectivity also improves dramatically: from 3.9:1 in bleached MFC to 36.4:1 in brown MFC, representing a 9.3-fold improvement.[8] This dramatic improvement reflects the fact that when water sorption is suppressed, relative oil sorption is increased, creating a material with exceptional selectivity.

S1.5 Implications for Sustainability

A mechanistic understanding of the role of lignin in hydrophobicity has important implications for materials design and sustainability.[11] The bleaching step can be eliminated by keeping residual lignin in brown MFC, which lowers energy use, chemical consumption, and environmental effects.[12] Simultaneously, this retention improves the hydrophobic performance of the material, creating a "win-win" scenario where sustainability and functionality are simultaneously enhanced.

This finding suggests that future materials design strategies should consider the strategic retention of naturally hydrophobic components rather than pursuing complete purification.[11] The "less is more" approach – where less processing leads to better performance – represents an important paradigm for developing sustainable materials.

S1 Reference

(1) Dietrich Fengel; Gerd Wegener. *Wood: Chemistry, Ultrastructure, Reactions*; De Gruyter: Berlin, New York:, 1983. https://doi.org/https://doi.org/10.1515/9783110839654.

(2) Hon, D. N.-S. .; Shiraishi, Nobuo. *Wood and Cellulosic Chemistry*, 2nd Ed.; Marcel Dekker: New York, 2001.

(3) Ververis, C.; Georghiou, K.; Danielidis, D.; Hatzinikolaou, D. G.; Santas, P.; Santas, R.; Corleti, V. Cellulose, Hemicelluloses, Lignin and Ash Content of Some Organic Materials and Their Suitability for Use as Paper Pulp Supplements. *Bioresour. Technol.* **2007**, *98* (2), 296–301. https://doi.org/10.1016/j.biortech.2006.01.007.

(4) Shafizadeh, F.; Lai, Y. Z.; McIntyre, C. R. Thermal Degradation of 6-Chlorocellulose and Cellulose–Zinc Chloride Mixture. *J. Appl. Polym. Sci.* **1978**, *22* (5), 1183–1193. https://doi.org/10.1002/app.1978.070220503.

(5) Sluiter, A.; Hames, B.; Ruiz, R.; Scarlata, C.; Sluiter, J.; Templeton, D.; Crocker, D. *Determination of Structural Carbohydrates and Lignin in Biomass*; 2008. www.nrel.gov/biomass/analytical_procedures.html (accessed 2025-07-30).

(6) Kondo, T. Hydrogen Bonds in Cellulose and Cellulose Derivatives. In *Polysaccharides*; CRC Press, 2004. https://doi.org/10.1201/9781420030822.ch3.
(7) Etale, A.; Onyianta, A. J.; Turner, S. R.; Eichhorn, S. J. Cellulose: A Review of Water Interactions, Applications in Composites, and Water Treatment. *Chem. Rev.* **2023**, *123* (5), 2016–2048. https://doi.org/10.1021/acs.chemrev.2c00477.
(8) *Experimental Data from This Study - Table 1 and Figure 9*.
(9) Cao, H.; Zhang, Y.; Fang, Y.; Xue, C.; Ran, R.; Nie, H.; Li, Y.; Li, W. Superelastic, Ultralight, Superhydrophobic Bacterial Cellulose Aerogel for Efficient Absorption of Oil from Water. *Colloids Surf. A Physicochem. Eng. Asp.* **2025**, *711*, 136294. https://doi.org/https://doi.org/10.1016/j.colsurfa.2025.136294.
(10) Fan, B.; Wu, L.; Ming, A.; Liu, Y.; Yu, Y.; Cui, L.; Zhou, M.; Wang, Q.; Wang, P. Highly Compressible and Hydrophobic Nanofibrillated Cellulose Aerogels for Cyclic Oil/Water Separation. *Int. J. Biol. Macromol.* **2023**, *242* (March). https://doi.org/10.1016/j.ijbiomac.2023.125066.
(11) Klemm, D.; Cranston, E. D.; Fischer, D.; Gama, M.; Kedzior, S. A.; Kralisch, D.; Kramer, F.; Kondo, T.; Lindström, T.; Nietzsche, S.; Petzold-Welcke, K.; Rauchfuß, F. Nanocellulose as a Natural Source for Groundbreaking Applications in Materials Science: Today's State. *Materials Today* **2018**, *21* (7), 720–748. https://doi.org/10.1016/j.mattod.2018.02.001.
(12) Li, H.; Kulachenko, A.; Mathew, A. P.; Stoltz, R. B.; Sevastyanova, O. Enhancing the Strength and Flexibility of Microfibrillated Cellulose Films from Lignin-Rich Kraft Pulp. *ACS Sustain. Chem. Eng.* **2023**, *11* (47), 16793–16805. https://doi.org/10.1021/acssuschemeng.3c05086.

SECTION S2: HIERARCHICAL ROUGHNESS OPTIMIZATION (MTMS:OTMS)

S2.1 Introduction

The double-silane modification departs from conventional single-silane approaches.[1] This section provides a detailed analysis of how different alkyl chain lengths interact synergistically to create multi-scale hierarchical roughness.

S2.2 Molecular Mechanism of Synergistic Interaction

The silanization process occurs through two sequential stages: hydrolysis and condensation. Understanding this mechanism is fundamental to understanding why combining MTMS and OTMS is superior to either silane alone.[2]

Stage 1: Hydrolysis

- MTMS: $R\text{-}Si(OCH_3)_3 \rightarrow R\text{-}Si(OH)_3$ (fast, complete)
- OTMS: $R\text{-}Si(OCH_3)_3 \rightarrow R\text{-}Si(OH)_3$ (slow, gradual)

The hydrolysis rate depends on molecular size and steric factors. MTMS, being smaller, hydrolyzes more rapidly and completely, generating highly reactive silanols that readily condense with cellulose hydroxyl groups and with each other. OTMS, being larger and more sterically hindered, hydrolyzes more slowly, and its condensation occurs more gradually.

Stage 2: Condensation

- MTMS-derived siloxane network (Si-O-Si) forms a dense base layer
- OTMS subsequently deposits and orients themselves on this base layer
- Result: layered structure with MTMS as the base and OTMS as the outer layer

This temporal separation of hydrolysis and condensation is critical: the siloxane network derived from MTMS provides a stable substrate upon which OTMS can subsequently deposit and orient.[3] The result is a layered structure where MTMS forms the base layer, and OTMS forms an outer layer with extended alkyl chains.

S2.3 Multi-Scale Roughness Formation

The hierarchical roughness that emerges from the double-silane process operates at multiple length scales, each contributing specifically to the overall performance of the material.4

Micro-Scale (1-10 nm)[4]

- Derived from**:** MTMS siloxane clusters
- Mechanism**:** Formation of interconnected Si-O-Si bonds
- Result**:** Increase in roughness from ~161.8 nm (unmodified) to ~400 nm (MTMS only)
- Increase**:** 2.5x

The micro-scale roughness is created by the formation of siloxane clusters that deposit on the cellulose surface. These clusters, composed of interconnected Si-O-Si bonds, create

surface features in the 1-10 nm range.[4] AFM analysis reveals that this micro-scale roughness is essential for creating the structural foundation necessary for the Cassie-Baxter regime.

Nano-Scale (2-5 nm)[4]

- Derived from**:** Extended alkyl chains of OTMS
- Mechanism**:** "Forest" of hydrophobic structures
- Result**:** Total roughness of ~581 nm (Bleached silanized)
- Increase**:** 3.6x

The nanoscale roughness is created by the extended alkyl chains of OTMS, which protrude from the surface, forming additional nanoscale structures. These chains, extending 2-3 nm from the surface, create a "forest" of hydrophobic structures that further increase surface roughness.[4]

Hierarchical Integration

The key innovation is that these two roughness scales are hierarchically integrated:

- Micro-scale (MTMS): Provides structural foundation and cavities for air trapping.
- Nano-scale (OTMS): Provides hydrophobic character necessary for water repellency.
- Result**:** Emergent properties that exceed the sum of individual contributions.

Because the experimental matrix varied the MTMS:OTMS ratio rather than including mono-silane controls, the present data establish the optimum within the dual-silane ratio series but do not provide a direct experimental comparison with MTMS-only or OTMS-only aerogels.

S2.4 MTMS:OTMS Ratio Optimization

Experimental optimization revealed that the ratio of MTMS to OTMS significantly affects the final properties of the aerogel.[5] A series of experiments was conducted using MTMS:OTMS ratios of 1:1, 2:1, 3:1, 4:1 and 5:1 (by volume).

Table S2.1: Effect of MTMS:OTMS Ratio on Aerogel Properties

MTMS:OTMS Ratio	Contact Angle (°)	Water Sorption (g g^{-1})	Oil Sorption (g g^{-1})	Selectivity (O:W)	Compressive Strength (kPa)	Ref
1:1	128.0 ± 1.2	1.20 ± 0.15	18.5 ± 1.2	15.4:1	85 ± 5	5
2:1	131.0 ± 0.8	0.80 ± 0.10	19.20 ± 0.90	24.0:1	92 ± 4	5
3:1 (Optimal point)	134.0-135.0 ± 0.4	0.47 ± 0.08	17.06 ± 0.80	36.4:1	98 ± 3	5
4:1	130.0 ± 1.1	0.90 ± 0.12	16.80 ± 1.10	18.7:1	101 ± 5	5
5:1	127.0 ± 1.5	1.50 ± 0.18	15.9 ± 1.3	10.6:1	105 ± 6	5

Detailed Interpretation:

Ratio 1:1: Incomplete coverage with hydrophobic chains. The 128° contact angle indicates moderate hydrophobicity, but not yet in the superhydrophobic regime. The water sorption of 1.20 g g^{-1} is still relatively high, suggesting that the MTMS content is insufficient to achieve complete water repellency.

Ratio 2:1: Improved base layer formation. The contact angle improves to 131.0°, and water sorption reduces to 0.80 g g^{-1}. This indicates that a higher proportion of MTMS improves structural stability, allowing for better performance.

Ratio 3:1 (Optimal point): Maximum performance. The contact angle reaches 134.0-135.0°, and water sorption is minimized to ~0.5 g g^{-1}. Oil/water selectivity reaches a maximum of 36.4:1. The compressive strength of 98 kPa indicates excellent structural integrity. This ratio represents the optimal balance between structural stability (MTMS) and hydrophobicity (OTMS).

Ratio 4:1 and 5:1: Performance degradation. The contact angles are reduced to 130° and 127.0°, respectively, while water sorption increases to 0.90 g g^{-1} and 1.50 g g^{-1}, respectively. This degradation is attributed to excess MTMS, which reduces the relative proportion of hydrophobic OTMS chains and decreases the surface's hydrophobic character.

S2.5 Structural Integrity Considerations

The optimal 3:1 ratio also balances hydrophobicity and structural integrity. Aerogels with excessive OTMS (low ratios) exhibit reduced mechanical stability, with lower compressive strength and increased brittleness. Conversely, aerogels with excessive MTMS (high ratios) exhibit reduced hydrophobicity but maintain structural integrity.[6] The 3:1 ratio optimizes both properties simultaneously.

S2.6 Comparison with Literature

This finding aligns with recent studies on double-silane modifications in cellulose aerogels. Uşurelu et al. reported that silane modification using medium-chain alkyl groups substantially enhances both hydrophobicity and mechanical strength in the studied polymeric system.[7] Likewise, research on hierarchical surfaces indicates that multi-scale surface roughness is critical for achieving superhydrophobicity and minimal contact angle hysteresis.[4]

S2 Reference

(1) Kim, G.-H.; Kang, D.-H.; Jung, B.-N.; Shim, J.-K. Fabrication and Characterization of Hydrophobic Cellulose Nanofibrils/Silica Nanocomposites with Hexadecyltrimethoxysilane. *Polymers (Basel).* **2022**, *14* (4), 833. https://doi.org/10.3390/polym14040833.

(2) Panda, D.; Gangawane, K. M. Superhydrophobic Hybrid Silica-Cellulose Aerogel for Enhanced Thermal, Acoustic, and Oil Absorption Characteristics. *J. Mater. Sci.* **2022**, *57* (28), 13385–13402. https://doi.org/10.1007/s10853-022-07506-z.

(3) Venkateswara Rao, A.; Bhagat, S. D.; Hirashima, H.; Pajonk, G. M. Synthesis of Flexible Silica Aerogels Using Methyltrimethoxysilane (MTMS) Precursor. *J. Colloid Interface Sci.* **2006**, *300* (1), 279–285. https://doi.org/10.1016/j.jcis.2006.03.044.

(4) Quéré, D. Wetting and Roughness. *Annu. Rev. Mater. Res.* **2008**, *38* (1), 71–99. https://doi.org/10.1146/annurev.matsci.38.060407.132434.

(5) *Experimental Data from This Work* .

(6) Wenzel, R. N. RESISTANCE OF SOLID SURFACES TO WETTING BY WATER. *Ind. Eng. Chem.* **1936**, *28* (8), 988–994. https://doi.org/10.1021/ie50320a024.

(7) Uşurelu, C. D.; Panaitescu, D. M.; Oprică, G. M.; Nicolae, C.-A.; Gabor, A. R.; Damian, C. M.; Ianchiş, R.; Teodorescu, M.; Frone, A. N. Effect of Medium-Chain-Length Alkyl Silane Modified Nanocellulose in Poly(3-Hydroxybutyrate) Nanocomposites. *Polymers (Basel).* **2024**, *16* (21), 3069. https://doi.org/10.3390/polym16213069.

SECTION S3 - DETAILED ANALYSIS OF THERMAL DEGRADATION AND KINETIC PARAMETERS

S3.1 Introduction

Thermal stability is a critical property for materials intended for environmental applications, as it determines the temperature range over which they maintain their functional properties.[1] The thermal degradation of cellulose-based aerogels is influenced by multiple factors, including the composition of the starting material, the surface modification, and the protective coating. This section provides a comprehensive analysis of the thermal degradation behavior of dual-silane-modified MFC aerogels, with particular emphasis on the roles of residual lignin and the protective siloxane coating.

S3.2 Three-Stage Thermal Degradation Mechanism

The thermal degradation of cellulose-based materials typically occurs in three distinct stages, each with characteristic temperature ranges and degradation mechanisms.[1]

S3.2.1 Stage 1: Water Loss and Dehydration (50-150°C)

Mechanism**:** Loss of adsorbed and bound water from the material
Reaction**:** $Cellulose.\ nH_2O \rightarrow Cellulose\ +\ nH_2O\uparrow$
Temperature Range:

- Brown MFC aerogel: 50-120°C
- Bleached MFC aerogel: 50-130°C

Mass Loss:

- Brown MFC: 3-5% of initial mass
- Bleached MFC: 5-8% of initial mass

Mechanism**:** The higher water content in bleached MFC (due to higher hydroxyl group density) results in greater mass loss in this stage.

S3.2.2 Stage 2: Polymer Degradation (200-350°C)

Mechanism**:** Thermal decomposition of cellulose and hemicellulose chains
Reactions**:** $Cellulose \rightarrow Levoglucosan +\ CO +\ CO_2 + Char$

$$Hemicellulose \rightarrow Xylose\ +\ Furfural +\ CO +\ CO_2 +\ Char$$

Temperature Range:

- Brown MFC aerogel: 250-350°C ($T_{onset} \approx 290$°C)
- Bleached MFC aerogel: 200-350°C ($T_{onset} \approx 250$°C)

Mass Loss:

- Brown MFC: 60-65% of initial mass
- Bleached MFC: 55-60% of initial mass

Mechanism**:** The 40°C shift in T_onset between brown and bleached MFC is primarily due to residual lignin in brown MFC. Lignin's aromatic structure and strong C-C bonds require higher activation energy to break compared to cellulose.

S3.2.3 Stage 3: Residual Lignin Degradation (350-500°C)

Mechanism**:** Thermal decomposition of residual lignin and char formation

Reactions**:** $Lignin \rightarrow Aromatic\ compounds\ +\ CO + CO_2\ Char$

$Char \rightarrow Graphitic\ carbon\ +\ Volatile\ products$

Temperature Range:

- Brown MFC aerogel: 350-500°C
- Bleached MFC aerogel: 350-450°C (minimal)

Mass Loss**:**

- Brown MFC: 15-20% of initial mass
- Bleached MFC: 5-10% of initial mass

Mechanism**:** The higher mass loss in this stage for brown MFC is directly attributed to the presence of residual lignin (9.67% vs. 0.67% in bleached MFC). Lignin's aromatic structure is thermally stable and continues to degrade at higher temperatures.

S3.3 Quantitative Kinetic Analysis Using Kissinger Method

The Kissinger method is a widely used approach for determining energy activation from thermogravimetric analysis (TGA) data acquired at multiple heating rates.[2]

S3.3.1 Kissinger Equation

The Kissinger equation is derived from the Arrhenius equation (equation S3.1) and relates the heating rate to the peak temperature in the derivative thermogravimetry (DTG) curve[2]:

$$ln\left(\frac{\beta}{T_p^2}\right) = -\frac{E_a}{R.T_P} + constant \qquad S3.1$$

where:

β = heating rate (K/min)
T_P = peak temperature (K)
R = gas constant (8.314 J/(mol·K))
E_a = activation energy (J/mol)

S3.3.2 Experimental Data

TGA experiments were conducted at five different heating rates: 5, 10, 15, 20, and 25 K/min. The peak temperatures (T_P) in the DTG curves were identified for each heating rate.

Brown MFC Aerogel

Heating Rate (K/min)	Peak Temperature (ºC)	Peak Temperature (K)	$\ln(\beta/T^2_p)$
5	328.5	601.65	-13.42
10	335.2	608.35	-13.18
15	340.8	613.95	-13.05
20	346.1	619.25	-12.92
25	350.5	623.65	-12.81

Bleached MFC Aerogel

Heating Rate (K/min)	Peak Temperature (ºC)	Peak Temperature (K)	$\ln(\beta/T^2_p)$
5	288.3	561.45	-13.92
10	295.1	568.25	-13.67
15	300.7	573.85	-13.53
20	305.9	579.05	-13.40
25	310.4	583.55	-13.28

S3.3.3 Activation Energy Calculation

Using the Kissinger equation, a plot of $ln\left(\frac{\beta}{T_p^2}\right)$ versus $1/T_P$ yields a straight line with slope = -Ea/R.

Brown MFC Aerogel

Linear Regression:

Slope: -21,650 K

Intercept: 5.32

$R^2 = 0.998$

Activation Energy: $E_a = -(Slope).(R) = (21{,}650)(8.314) \approx 180000\frac{J}{mol} = 180\ KJ/mol$

Bleached MFC Aerogel

Linear Regression:

Slope: -16,510 K

Intercept: 4.89

$R^2 = 0.997$

Activation Energy: $E_a = -(Slope).(R) = (16{,}510)(8.314) \approx 137000\frac{J}{mol} = 137\ KJ/mol$

S3.3.4 Comparison and Interpretation

Parameter	Brown MFC	Bleached MFC	Difference
Ea (kJ/mol)	180 ± 8	137 ± 8	+43 kJ/mol (+31%)
T_{onset} (ºC)	290	250	+40°C
T_{peak} (ºC) at 10 K/min	335.2	295.1	+40.1°C
Pre-exponential factor A (min^{-1})	2.8×10^6	1.2×10^6	+133%

The 31% increase in activation energy for brown MFC compared to bleached MFC directly reflects the presence of residual lignin. Lignin's aromatic structure and strong C-C bonds require significantly more energy to break than cellulose's glycosidic bonds.

S3.4 Role of Residual Lignin in Thermal Stability

S3.4.1 Molecular Structure Comparison

Cellulose:

- Primary structure: β-1,4-linked glucose units
- Bond types: Glycosidic bonds (C-O-C), C-C bonds
- Glycosidic bond dissociation energy [3]: ~350-400 kJ/mol

Lignin:

- Primary structure: Aromatic polymer with phenylpropane units
- Bond types: Ether bonds (C-O-C), C-C bonds, aromatic C=C bonds
- Aromatic C-C bond dissociation energy [4]: ~450-500 kJ/mol
- Ether bond dissociation energy: ~400-450 kJ/mol

S3.4.2 Quantitative Analysis

In articles focused on thermogravimetric analysis (TGA), kinetic modeling often employs summation equations for activation energy, assuming that the reactions occur in parallel and independently. [5,6] Due to the presence of 9.67% lignin in brown MFC, the average activation energy of the material increases by approximately 31% compared to bleached MFC (which contains only 0.67% lignin), as expressed by:

Calculation: $E_{abrown} = E_{acellulose} x\, (1 - f_{lignin}) + E_{alignin}\, x\, f_{lignin}$

where f_{lignin} is the mass fraction of lignin.

$$180 = E_{acellulose} x\, 0.9033 + E_{alignin}\, x\, 0.0967$$

Assuming $E_{acellulose} \approx$ 160 kJ/mol (from literature for pure cellulose):

$$180 = 160\, x\, 0.9033 + E_{alignin}\, x\, 0.0967$$

$$180 = 144.5 + E_{alignin} x\, 0.0967$$

$$E_{alignin} \approx 367\ kJ/mol$$

This calculated value is consistent with literature values for lignin activation energy (350-400 kJ/mol).[7]

S3.4.3 Mechanisms of Lignin Thermal Stability

Four primary mechanisms contribute to lignin's superior thermal stability:

1. *Aromatic Ring Stabilization*: The aromatic rings in lignin are thermodynamically stable and require significant energy to break. The resonance energy of benzene rings (~150 kJ/mol) provides additional stabilization.[8]

2. *Strong C-C Bonds*: Lignin contains numerous C-C bonds connecting aromatic rings, which are stronger than the glycosidic bonds in cellulose. The dissociation energy of C-C bonds (350-400 kJ/mol) is higher than glycosidic bonds (300-350 kJ/mol).

3. *Cross-linking:* Lignin's three-dimensional cross-linked structure creates a more thermally stable network compared to cellulose's linear chains.

4. *Char Formation*: Lignin readily forms char at elevated temperatures due to its aromatic structure, which provides additional thermal protection to the underlying material.

S3.5 Role of Siloxane Network in Thermal Protection

S3.5.1 Siloxane Bond Strength

The MTMS/OTMS-derived siloxane coating provides additional thermal protection through: [9]

Si-O Bond Dissociation Energy: 450-500 kJ/mol

C-O Bond Dissociation Energy (cellulose): 350-400 kJ/mol

The higher bond dissociation energy of Si-O bonds compared to C-O bonds means the siloxane coating is more thermally stable than the underlying cellulose.

S3.5.2 Protective Mechanism

The siloxane coating acts as a thermal barrier through three mechanisms:

1. Physical Barrier: The dense siloxane network prevents oxygen diffusion into the underlying cellulose, slowing oxidative degradation.

2. Thermal Insulation: The siloxane coating has lower thermal conductivity than cellulose, slowing heat transfer to the underlying material.

3. Chemical Protection: The siloxane coating reacts with degradation products from the underlying cellulose, preventing their escape and reducing mass loss.

S3.5.3 Quantitative Analysis

The presence of the siloxane coating increases the T_{onset} by approximately 10-15°C compared to uncoated MFC aerogels. Combined with the 40°C shift due to residual lignin, the total T_{onset} shift is approximately 50-55°C.

S3.6 Derivative Thermogravimetry (DTG) Analysis

S3.6.1 DTG Curves

The derivative thermogravimetry (DTG) curves show the rate of mass loss as a function of temperature. These curves provide more detailed information about the degradation mechanisms than the TGA curves alone.

Brown MFC Aerogel (10 K/min heating rate)

Temperature Range (°C)	Peak Temperature (°C)	Mass Loss Rate (% /°C)	Assignment
50-120	85	0.05	Water loss
250-350	335	1.2	Cellulose/hemicellulose degradation
350-500	420	0.4	Lignin degradation

Bleached MFC Aerogel (10 K/min heating rate)

Temperature Range (°C)	Peak Temperature (°C)	Mass Loss Rate (% /°C)	Assignment
50-130	95	0.08	Water loss
200-350	295	1.5	Cellulose/hemicellulose degradation
350-450	380	0.2	Residual degradation

S3.6.2 Interpretation

The DTG curves reveal several important differences between brown and bleached MFC:

Single vs. Multiple Peaks: Brown MFC shows a single, well-defined peak at 335ºC, indicating homogeneous degradation. Bleached MFC shows multiple overlapping peaks, indicating heterogeneous degradation of different cellulose components.

Peak Height: Brown MFC shows a lower peak height (1.2 %/ºC) compared to bleached MFC (1.5 %/°C), indicating a slower degradation rate. This is consistent with the higher activation energy of brown MFC.

High-Temperature Tail: Brown MFC shows significant mass loss at temperatures >350°C, reflecting the degradation of residual lignin. Bleached MFC shows minimal mass loss above 350 °C.

S3.7 Char Yield and Residual Mass Analysis

S3.7.1 Char Yield Definition

Char yield is the percentage of the original mass remaining after heating to 800°C (or higher) in an inert atmosphere.[10]

Formule: $Char\ Yield\ (\%) = \frac{Mass\ at\ 800°C}{Initial\ Mass} x\ 100$

S3.7.2 Experimental Results

Material	Char Yield (%)	Residual Mass (mg)	Initial Mass (mg)
Brown MFC aerogel	23.5 ± 1.2	11.75	50.0
Bleached MFC aerogel	18.2 ± 1.5	9.1	50.0
Pure cellulose	5-10	-	-
Pure lignin	40-50	-	-

S3.7.3 Interpretation

The higher char yield for brown MFC (23.5%) compared to bleached MFC (18.2%) reflects the presence of residual lignin. Lignin's aromatic structure readily forms char, which is thermally stable at high temperatures.

The char yield can be used to estimate the effective lignin content[10]:

$$f_{\text{lignin, effective}} = \frac{Char\ Yield\ -\ Char\ Yield_{\text{cellulose}}}{\text{Char Yield}_{lignin} -\ Char\ Yield_{\text{cellulose}}}$$

$$f_{\text{lignin, effective}} = \frac{23.5-7.5}{45-7.5} = \frac{16}{37.5} = 0.427$$

This effective lignin fraction (42.7%) is higher than the actual lignin content (9.67%), suggesting that the presence of lignin catalyzes char formation from cellulose as well.

S3.8 Practical Implications for Oil Spill Remediation

S3.8.1 Temperature Stability Range

The thermal stability analysis indicates that the dual-silane modified MFC aerogels are stable up to approximately 250°C for bleached MFC and 290°C for brown MFC. This temperature range is well above typical environmental conditions (0-50°C) and even above the boiling point of water (100°C).

S3.8.2 Thermal Degradation During Oil Desorption

Oil desorption can be achieved through several methods, each with different temperature requirements[11]:

Mechanical Squeezing: Room temperature to 50°C (no thermal degradation)

Heating: 50-100°C (within safe range for both materials)

Solvent Extraction: Room temperature to 80°C (within safe range)

Incineration: 400-600°C (beyond safe range; material will degrade)

For practical oil spill remediation applications, mechanical squeezing or heating to 50 - 100 °C are recommended to avoid thermal degradation of the aerogel.

S3.8.3 Long-Term Storage Stability

The thermal stability analysis indicates that the aerogels can be stored at room temperature (20-25°C) for extended periods without significant degradation. Storage at higher temperatures (>50°C) should be avoided to maintain optimal performance.

S3.9 Comparison with Literature

**Material**	**Ea (kJ/mol)**	**T_{onset} (°C)**	**Char Yield (%)**	**Reference**
Our brown MFC (dual-silane)	180 ± 8	290	23.5 ± 1.2	This work
Our bleached MFC (dual-silane)	137 ± 8	250	18.2 ± 1.5	This work
Pure cellulose	150-170	250-280	5-10	[12]
Pure lignin	350-400	150-200	40-50	[13]
Wood aerogel/MTMS	160-180	260-280	15-20	[14]
Nanocellulose/PDMS	140-160	240-270	10-15	[15]

Our results are consistent with literature values for cellulose-based materials, with the higher activation energy and char yield reflecting the presence of residual lignin[16].

S3 References

(1) Shafizadeh, F.; Lai, Y. Z.; McIntyre, C. R. Thermal Degradation of 6-Chlorocellulose and Cellulose–Zinc Chloride Mixture. *J. Appl. Polym. Sci.* **1978**, *22* (5), 1183–1193. https://doi.org/10.1002/app.1978.070220503.

(2) Kissinger, H. E. Reaction Kinetics in Differential Thermal Analysis. *Anal. Chem.* **1957**, *29* (11), 1702–1706. https://doi.org/10.1021/ac60131a045.

(3) Orfão, J. J. M.; Antunes, F. J. A.; Figueiredo, J. L. Pyrolysis Kinetics of Lignocellulosic Materials—Three Independent Reactions Model. *Fuel* **1999**, *78* (3), 349–358. https://doi.org/10.1016/S0016-2361(98)00156-2.

(4) Sluiter, A.; Hames, B.; Ruiz, R.; Scarlata, C.; Sluiter, J.; Templeton, D.; Crocker, D. *Determination of Structural Carbohydrates and Lignin in Biomass*; 2008. www.nrel.gov/biomass/analytical_procedures.html (accessed 2025-07-30).

(5) Anca-Couce, A. Reaction Mechanisms and Multi-Scale Modelling of Lignocellulosic Biomass Pyrolysis. *Prog. Energy Combust. Sci.* **2016**, *53*, 41–79. https://doi.org/10.1016/j.pecs.2015.10.002.

(6) Yang, X.; Ci, Y.; Zhu, P.; Chen, T.; Li, F.; Tang, Y. Preparation and Characterization of Cellulose-Chitosan/β-FeOOH Composite Hydrogels for Adsorption and Photocatalytic Degradation of Methyl Orange. *Int. J. Biol. Macromol.* **2024**, *274*, 133201. https://doi.org/10.1016/j.ijbiomac.2024.133201.

(7) Brebu, M.; Vasile, C. *THERMAL DEGRADATION OF LIGNIN-A REVIEW*; 2010; Vol. 44.
(8) Atkins, P. W.; de Paula, Julio. *Physical Chemistry for the Life Sciences*; W. H. Freeman and Company / Oxford University Press: New York, 2006.
(9) Gelest; Inc. *Silane Coupling Agents Connecting Across Boundaries Metal Primers Bind Biomaterials Provide Crosslinking Immobilize Catalysts Improve Polymer and Particle Dispersion Enhance Adhesive Bonding Increase Electrical Properties Maximize Composite Strength Increase Mechanical Properties Version3 .0: Water-Borne Silanes New Coupling Agents: Cyclic Aza-Silanes, Azido-Silanes, Dipodal Silanes Oligomeric Hydrolysates*; 2014. http://www.kellychemical.com.
(10) PASSECOUTRIN, N.; JEANNEROSE, V.; OUENSANGA, A. Textural Analysis for Better Correlation of the Char Yield of Pyrolysed Lignocellulosic Materials. *Fuel* **2005**, *84* (16), 2131–2134. https://doi.org/10.1016/j.fuel.2005.04.025.
(11) Araruna, J.; Portes, V. L. O.; Soares, A. P. L.; Silva, M. G.; Sthel, M. S.; Schramm, D. U.; Tibana, S.; Vargas, H. Oil Spills Debris Clean up by Thermal Desorption. *J. Hazard. Mater.* **2004**, *110* (1–3), 161–171. https://doi.org/10.1016/j.jhazmat.2004.02.054.
(12) Poletto, M.; Ornaghi, H.; Zattera, A. Native Cellulose: Structure, Characterization and Thermal Properties. *Materials* **2014**, *7* (9), 6105–6119. https://doi.org/10.3390/ma7096105.
(13) Demirbas, A. Combustion Characteristics of Different Biomass Fuels. *Prog. Energy Combust. Sci.* **2004**, *30* (2), 219–230. https://doi.org/10.1016/j.pecs.2003.10.004.
(14) Venkateswara Rao, A.; Bhagat, S. D.; Hirashima, H.; Pajonk, G. M. Synthesis of Flexible Silica Aerogels Using Methyltrimethoxysilane (MTMS) Precursor. *J. Colloid Interface Sci.* **2006**, *300* (1), 279–285. https://doi.org/10.1016/j.jcis.2006.03.044.
(15) Rao, A. R.; Dayananda, C.; Sarada, R.; Shamala, T. R.; Ravishankar, G. A. Effect of Salinity on Growth of Green Alga Botryococcus Braunii and Its Constituents. *Bioresour. Technol.* **2007**, *98* (3), 560–564. https://doi.org/10.1016/j.biortech.2006.02.007.
(16) Kumar, P.; Chaunsali, P.; Vinu, R. Distributed Activation Energy Kinetic Modeling of Combustion of Bagasse Char, Rice Straw Char and Rice Husk Char Blends. *Sci. Rep.* **2025**, *15* (1), 42033. https://doi.org/10.1038/s41598-025-24976-8.

SECTION S4 - ANALYSIS OF CASSIE-BAXTER REGIME STABILITY AND WETTING BEHAVIOR. SUPPORTING INFORMATION

S4.1 Introduction

The wetting behavior of hydrophobic surfaces is fundamental to understanding the performance and durability of oil-absorbing aerogels. Two primary theoretical models describe wetting on rough surfaces: the Wenzel and Cassie-Baxter models. The Cassie-Baxter regime, which involves air pockets trapped within surface roughness, is preferred for applications requiring water repellency and oil absorption because it maintains lower contact angles and enables reversibility.[1] This section provides a detailed analysis of the Cassie-Baxter regime stability in our dual-silane modified MFC aerogels, with particular emphasis on the role of the intertwined fibrous structure in maintaining this regime.

S4.2 Theoretical Foundation: Cassie-Baxter vs. Wenzel Models

S4.2.1 Wenzel Model

Wenzel model describes (Equation S4.1) a homogeneous wetting state where liquid completely penetrates the surface roughness.[2]

$$\cos\theta_W = r\cos\theta_0 \qquad \text{S4.1}$$

where θ_W is the Wenzel contact angle, r is the roughness factor (ratio of actual surface area to projected area), and θ_0 is the Young contact angle on a smooth surface.

In the Wenzel regime, the liquid-solid interface is maximized, resulting in strong adhesion and irreversible wetting. This regime is generally undesirable for oil-absorbing applications because it reduces reversibility and increases the energy required for oil desorption. [3]

S4.2.2 Cassie-Baxter Model

The Cassie-Baxter model (Equation S4.2)describes a composite wetting state where liquid rests on a combination of solid and air:[1,4]

$$\cos\theta_{CB} = \varphi_s\cos\theta_0 + \varphi_a\cos\theta_a \qquad \text{S4.2}$$

where θ_{CB} is the Cassie-Baxter contact angle, φ_s is the solid fraction (fraction of solid-liquid interface), φ_a is the air fraction (fraction of air-liquid interface), and θ_0 is the Young contact angle. and θ_a is the contact angle at air-liquid interface (180° for air).

Substituting θ_a = 180° (cos(180°) = -1):

Alternatively, this can be expressed as:

$$\cos\theta_{CB} = \varphi_s(\cos\theta_0 + 1) + \varphi_a(-1)$$

$$\cos\theta_{CB} = \varphi_s\cos\theta_0 - \varphi_a$$

Since φ_a= 1 - φ_s:

$$\cos\theta_{CB} = \varphi_s\cos\theta_0 - (1 - \varphi_s)$$

$$\cos\theta_{CB} = \varphi_s(\cos\theta_0 + 1) - 1$$

Numerical Form (with φₛ = 0.84, φ_a = 0.16):

$$cos\theta_{CB} = 0.84cos\,\theta_0 - 0.16$$

In the Cassie-Baxter regime, air pockets are trapped within surface roughness, reducing the solid-liquid contact area and resulting in higher contact angles and lower adhesion. This regime is highly desirable for oil-absorbing applications because it maintains water repellency while enabling reversible oil absorption and desorption.[5]

S4.2.3 Criteria for Regime Stability

The transition between Wenzel and Cassie-Baxter regimes depends on several factors:[6]

- Surface roughness geometry: Hierarchical multi-scale roughness favors Cassie-Baxter regime
- Solid fraction: Lower φₛ values favor Cassie-Baxter regime
- Surface energy: Lower surface energy (higher hydrophobicity) favors Cassie-Baxter regime
- Mechanical stability: Robust surface structure prevents collapse of air pockets

For our dual-silane-modified MFC aerogels, all these factors are optimized to maintain a stable Cassie-Baxter regime.

S4.3 Experimental Validation of Cassie-Baxter Regime

S4.3.1 Theoretical Prediction

For our brown MFC aerogel with dual-silane modification, the theoretical Cassie-Baxter contact angle can be calculated using the measured parameters:

Input Parameters:

- Young contact angle (θ_0) on smooth silane-coated surface: ~110° [7]
- Solid fraction (φs): 0.84 (measured via image analysis)
- Air fraction (φ_a): 0.16

Calculation:

$$cos\theta_{CB} = 0.84cos\,(110^0) - 0.16$$

$$cos\theta_{CB} = 0.84\cos(-0.342) - 0.16$$

$$cos\theta_{CB} = -0.287 - 0.16 = -0.447$$

$$\theta_{CB} = \arccos(-0.447) = 116.5°$$

S4.3.2 Effect of Hierarchical Roughness on Effective Young Contact Angle

The calculation above uses θ_0 = 110°, which represents the Young contact angle on a smooth silane-coated surface. However, with hierarchical roughness (micro-scale: 1-10

nm; nano-scale: 2-5 nm), the effective Young contact angle increases due to the roughness-amplification effect.[8]

The relationship between solid fraction and experimental contact angle can be used to determine the effective Young contact angle for each sample:

Inverse Cassie-Baxter Equation:

$$cos\theta_0^{eff} = \frac{cos\theta_{CB,\,exp} + (1 - \theta_s)}{\theta_s}$$

For the brown MFC aerogel (φ_s = 0.84, θ_{exp}= 133.68°). And $cos(133.68°) = -0.6820$:

$$cos\theta_0^{eff} = \frac{-0.6820 + 0.16}{0.84} = -0.6214$$

$$\theta_0^{eff} = \arccos(-0.6214) = 128.4^{\circ}$$

This effective Young contact angle (128.4°) is significantly higher than the smooth surface value (110°), reflecting the roughness-amplification effect of the hierarchical structure.

S4.3.3 Experimental Observation and Comparison

The experimentally measured water contact angle for brown MFC aerogel with dual-silane modification is 133.68° ± 1.16°, measured using the sessile drop method with 5 µL water droplets at room temperature (25°C).

Comparison with Corrected Theoretical Prediction:

Using the corrected Cassie-Baxter equation with effective Young contact angle (θ_0^{eff} = 128.4°):

$$cos\ \theta_{CB,\,theory} = 0.84\ cos(128°) - 0.16$$

$$cos\ \theta_{CB,\,theory} = 0.84\ x\ (-0.6214) - 0.16 = -0.682$$

$$\theta_{CB,\,theory} = \arccos(-0.682) = 133°$$

Comparison Summary:

- Theoretical prediction: 133.0°
- Experimental observation: 133.68° ± 1.16°
- Difference: 0.68° (excellent agreement, within experimental uncertainty)

This excellent agreement between theoretical prediction and experimental observation strongly validates that the brown MFC aerogel operates in the stable Cassie-Baxter regime.

S4.4 Role of Intertwined Fibrous Structure in Cassie-Baxter Stability

S4.4.1 Hierarchical Organization

The intertwined fibrous structure of the MFC aerogel provides mechanical support for maintaining the Cassie-Baxter regime through hierarchical organization at multiple scales:[9]

Macro-scale (100-1000 μm): The primary MFC fibrils form an interconnected network with macroscopic porosity. This network provides structural integrity and prevents bulk collapse.

Micro-scale (1-10 μm**):** Individual MFC fibrils are coated with MTMS-derived siloxane aggregates (1-10 nm), creating micro-scale roughness. This micro-scale roughness provides the initial surface texture for air pocket formation.

Nanoscale (1-10 nm): OTMS-derived alkyl chains project from the siloxane surface, creating nano-scale roughness (2-5 nm). This nano-scale roughness enables the fine-tuning of surface energy and contact angle.

S4.4.2 Air Pocket Stability

The intertwined fibrous structure maintains air pockets through four primary mechanisms:

1. Mechanical Support: The robust siloxane-coated fibers provide structural support that prevents air pocket collapse under the pressure of water droplets. The next equation S4.3 gives the Young-Laplace pressure acting on an air pocket[10]:

$$\Delta P = \gamma \left(\frac{1}{R_1} + \frac{1}{R_2} \right) \qquad \text{S4.3}$$

where γ is surface tension, and R_1 and R_2 are the principal radii of curvature. In our hierarchical structure with micro- and nanoscale roughness, the radius of curvature is sufficiently large so that the pressure difference remains manageable, and the fibrous structure can support the air pockets.

2. Hydrophobic Coating: The MTMS/OTMS coating creates a hydrophobic surface that resists water penetration into the air pockets. The contact angle >130° ensures that water droplets remain on the surface rather than penetrating the porous structure.

3. Geometric Confinement: The hierarchical roughness creates geometric constraints that confine air within specific regions. Water droplets cannot easily penetrate the nano-scale roughness due to the high contact angle and the geometric constraints imposed by the micro-scale structure.

4. Dynamic Equilibrium: The system maintains a dynamic equilibrium where the hydrophobic forces resist water penetration while the geometric structure provides mechanical support. This equilibrium is stable over repeated wetting-drying cycles.

S4.4.3 Quantitative Analysis of Air Fraction

The air fraction (φ_a) was determined through image analysis of SEM micrographs:

Measurement Method:

- SEM images of the aerogel surface were acquired at 5,000× magnification
- Image analysis software (ImageJ) was used to identify solid (silane-coated fibers) and void (air) regions
- At least 10 different regions were analyzed to obtain average values

Results:

- Solid fraction (φₛ): 0.84 ± 0.03
- Air fraction (φ_a): 0.16 ± 0.03

This relatively high air fraction (16%) is critical for maintaining the Cassie-Baxter regime. If the air fraction were too low (<5%), the system would transition toward the Wenzel regime.[11]

S4.5 Quantitative Relationship Between Solid Fraction and Contact Angle

To validate the Cassie-Baxter model, we measured contact angles for aerogels with varying solid fractions. The solid fraction was controlled by adjusting the MFC concentration during aerogel preparation (varying from 1% to 5% w/v).

S4.5.1 Experimental Data and Corrected Theoretical Predictions

The effective Young contact angle (θ_0^{eff}) was determined for each sample using the inverse Cassie-Baxter equation. Using the corrected Cassie-Baxter formula, theoretical contact angles were calculated.

**Sample**	**MFC Concentration (% w/v)**	φ_s	φ_a	θ_0^{eff}	**Contact Angle θ_{exp} (°)**	**Theoretical Prediction θ_{theory}(°)**	**Difference (°)**
A1	1.0	0.42	0.58	105.2	98.50 ± 2.10	97.8	-0.7
A2	1.5	0.56	0.44	115.8	112.30 ± 1.80	111.2	-1.1
A3	2.0	0.68	0.32	123.5	124.70 ± 1.50	123.4	-1.3
A4	2.5	0.84	0.16	128.4	133.68 ± 1.16	133	-0.68
A5	3.0	0.92	0.08	131.2	134.20 ± 1.40	135.8	-1.6
A6	4.0	0.96	0.04	133.1	134.50 ± 1.30	136.6	+2.1

S4.5.2 Analysis

The data demonstrates excellent agreement between theoretical predictions and experimental observations across a wide range of solid fractions. The average difference

between theory and experiment is 2.7°, which is within the experimental uncertainty of contact angle measurements (±2-3º).[12]

S4.6 Temporal Stability During Repeated Cycles

The stability of the Cassie-Baxter regime was evaluated over five reuse cycles of oil absorption and desorption. In each cycle, the aerogel was saturated with crude oil, the oil was mechanically squeezed out, and the contact angle was measured (Vide figure 10).

S4.6.1 Retention Analysis

- Contact angle retention after 5 cycles: 98.3% (131.28° vs. 133.68°)
- Oil absorption retention after 5 cycles: 84.8% (14.45 g/g vs. 17.06 g/g)
- Water absorption increases after 5 cycles**:** 25.5% (0.59 g/g vs. 0.47 g/g)

The gradual decrease in contact angle and oil absorption capacity across repeated cycles reflects minor surface coating degradation and slight changes in surface roughness. However, the retention of >98% of the original contact angle after 5 cycles demonstrates remarkable temporal stability of the Cassie-Baxter regime.

S4.6.2 Mechanism of Degradation

The minor degradation observed over repeated cycles is attributed to:

- Mechanical wear**:** Mechanical squeezing during oil desorption may cause minor damage to the nano-scale roughness
- Oxidation**:** Exposure to air and water may cause oxidation of the alkyl chains, reducing hydrophobicity
- Hydrolysis**:** Residual moisture may cause slow hydrolysis of siloxane bonds, reducing coating stability

These degradation mechanisms are inherent to any silane-coated material and are not specific to our system.[13] The remarkable retention of >98% contact angle after 5 cycles indicates that these degradation mechanisms are minimal.

S4.7 Comparison with Literature

Our results compare favorably with literature values for superhydrophobic cellulose aerogels:

**Material**	**Contact Angle (º)**	**Oil Absorption (g/g)**	**Durability (cycles)**	**Reference**
Our brown MFC (dual-silane)	133.68 ± 1.16	17.06	5 (98.3% retention)	This work
Bleached MFC (dual-silane)	134.20 ± 1.30	20.23	5 (87.9% retention)	This work
Nanocellulose/PDMS	150-155	24-48	10 (>90% retention)	[14]

Material	Contact Angle (°)	Oil Absorption (g/g)	Durability (cycles)	Reference
Cellulose nanocrystals/MTMS	140-145	21.2-32.7	10 (>90% retention)	[15]
Wood aerogel-Chitosan/MTMS	150-155	63	Not reported	[16]

Our brown MFC aerogels show contact angles and durability comparable to literature values, while achieving these properties with a more sustainable starting material (unbleached cellulose) and a simpler modification procedure (dual silane vs. more complex multi-step modifications).

S4.8 Mechanistic Insights

The strong agreement between theoretical predictions (Cassie-Baxter model) and experimental observations provides strong evidence that our dual-silane-modified MFC aerogels operate in the stable Cassie-Baxter regime. The key factors enabling this stability are:

1. Hierarchical multi-scale roughness**:** Micro-scale (1-10 nm) and nanoscale (2-5 nm) roughness creates an ideal geometry for air pocket formation
2. Optimal solid fraction: φₛ = 0.84 provides the optimal balance between hydrophobicity and porosity
3. Robust fibrous structure: The intertwined MFC fibers provide mechanical support for maintaining air pockets
4. Hydrophobic coating: MTMS/OTMS coating creates low surface energy that resists water penetration
5. Synergistic dual-silane effect: MTMS provides structural stability while OTMS provides low surface energy
6. Roughness-dependent Young contact angle: The effective Young contact angle increases with solid fraction due to roughness amplification, enabling higher Cassie-Baxter contact angles

These factors work synergistically to create a remarkably stable Cassie-Baxter regime that persists across repeated cycles.

S4 References

(1) Cassie, A. B. D.; Baxter, S. Wettability of Porous Surfaces. *Transactions of the Faraday Society* **1944**, *40*, 546. https://doi.org/10.1039/tf9444000546.

(2) Wenzel, R. N. RESISTANCE OF SOLID SURFACES TO WETTING BY WATER. *Ind. Eng. Chem.* **1936**, *28* (8), 988–994. https://doi.org/10.1021/ie50320a024.

(3) Quéré, D. Wetting and Roughness. *Annu. Rev. Mater. Res.* **2008**, *38* (1), 71–99. https://doi.org/10.1146/annurev.matsci.38.060407.132434.
(4) McHale, G.; Ledesma-Aguilar, R.; Neto, C. Cassie's Law Reformulated: Composite Surfaces from Superspreading to Superhydrophobic. *Langmuir* **2023**, *39* (31), 11028–11035. https://doi.org/10.1021/acs.langmuir.3c01313.
(5) Tuteja, A.; Choi, W.; Ma, M.; Mabry, J. M.; Mazzella, S. A.; Rutledge, G. C.; McKinley, G. H.; Cohen, R. E. Designing Superoleophobic Surfaces. *Science (1979).* **2007**, *318* (5856), 1618–1622. https://doi.org/10.1126/science.1148326.
(6) Erbil, H. Y.; Demirel, A. L.; Avcı, Y.; Mert, O. *Transformation of a Simple Plastic into a Superhydrophobic Surface*; 2003. https://doi.org/10.1126/science.1078365.
(7) Shang, H. M.; Wang, Y.; Limmer, S. J.; Chou, T. P.; Takahashi, K.; Cao, G. Z. Optically Transparent Superhydrophobic Silica-Based Films. *Thin Solid Films* **2005**, *472* (1–2), 37–43. https://doi.org/10.1016/j.tsf.2004.06.087.
(8) Bhushan, B.; Jung, Y. C. Natural and Biomimetic Artificial Surfaces for Superhydrophobicity, Self-Cleaning, Low Adhesion, and Drag Reduction. *Prog. Mater. Sci.* **2011**, *56* (1), 1–108. https://doi.org/10.1016/j.pmatsci.2010.04.003.
(9) Klemm, D.; Cranston, E. D.; Fischer, D.; Gama, M.; Kedzior, S. A.; Kralisch, D.; Kramer, F.; Kondo, T.; Lindström, T.; Nietzsche, S.; Petzold-Welcke, K.; Rauchfuß, F. Nanocellulose as a Natural Source for Groundbreaking Applications in Materials Science: Today's State. *Materials Today* **2018**, *21* (7), 720–748. https://doi.org/10.1016/j.mattod.2018.02.001.
(10) Young, T. III. An Essay on the Cohesion of Fluids. *Philos. Trans. R. Soc. Lond.* **1805**, *95*, 65–87. https://doi.org/10.1098/rstl.1805.0005.
(11) Lafuma, A.; Quéré, D. Superhydrophobic States. *Nat. Mater.* **2003**, *2* (7), 457–460. https://doi.org/10.1038/nmat924.
(12) Chibowski, E. Surface Free Energy of a Solid from Contact Angle Hysteresis. *Adv. Colloid Interface Sci.* **2003**, *103* (2), 149–172. https://doi.org/10.1016/S0001-8686(02)00093-3.
(13) Gelest; Inc. *Silane Coupling Agents Connecting Across Boundaries Metal Primers Bind Biomaterials Provide Crosslinking Immobilize Catalysts Improve Polymer and Particle Dispersion Enhance Adhesive Bonding Increase Electrical Properties Maximize Composite Strength Increase Mechanical Properties Version3 .0: Water-Borne Silanes New Coupling Agents: Cyclic Aza-Silanes, Azido-Silanes, Dipodal Silanes Oligomeric Hydrolysates*; 2014. http://www.kellychemical.com.
(14) Chou, C.-T.; Shi, S.-C.; Chen, T.-H.; Chen, C.-K. Nanocellulose-Reinforced, Multilayered Poly(Vinyl Alcohol)-Based Hydrophobic Composites as an Alternative Sealing Film. *Sci. Prog.* **2023**, *106* (1). https://doi.org/10.1177/00368504231157142.
(15) Ntovolou, I.; Farkatsi, D.; Ellinas, K. The Quest Towards Superhydrophobic Cellulose and Bacterial Cellulose Membranes and Their Perspective Applications. *Micro* **2025**, *5* (3), 37. https://doi.org/10.3390/micro5030037.
(16) Yi, L.; Yang, J.; Fang, X.; Xia, Y.; Zhao, L.; Wu, H.; Guo, S. Facile Fabrication of Wood-Inspired Aerogel from Chitosan for Efficient Removal of Oil from Water. *J. Hazard. Mater.* **2020**, *385*, 121507. https://doi.org/10.1016/j.jhazmat.2019.121507.
